# A Hybrid 2D–3D RGB-D Method for Growth Axis Estimation and Robotic Grasp Pose Generation of Phalaenopsis Orchid Buds

**DOI:** 10.1016/j.mex.2026.104104

**Published:** 2026-08-19

**Authors:** Tien-Duc Nguyen, Minh-Hieu Pham, Minh-Hau Le, Hoang-Hiep Ly, Xuan-Thuan Nguyen, Thi-Thoa Mac, Hong-Hai Hoang

**Affiliations:** a School of Mechanical Engineering, Hanoi University of Science and Technology, No. 1 Dai Co Viet Street, Hanoi, 100000, Vietnam; b Nam Dinh University of Technology Education, Phu Nghia Street, Thien Truong Ward, Ninh Binh Province, Vietnam

**Keywords:** RGB-D perception, skeleton-guided fusion, point cloud processing, plant phenotyping, principal component analysis, agricultural manipulation, grasp planning

## Abstract

This article presents a reproducible hybrid 2D–3D RGB-D method for estimating the growth axis of Phalaenopsis orchid buds and converting it into a robotic grasp pose. Synchronized RGB images and depth maps are used as input. Instance segmentation first isolates individual buds, and the resulting masks are used for 2D skeleton extraction and mask-constrained 3D point cloud reconstruction. After point cloud denoising, background plane removal, and coordinate normalization, branch-wise Principal Component Analysis estimates local geometric direction candidates. These candidates are fused with skeleton-derived branch topology to determine the final growth axis. The estimated axis is then used to construct a local grasp-oriented coordinate frame and generate a 6-DoF grasp pose in the robot base frame through camera–robot calibration. The method was evaluated on 520 RGB-D samples of Phalaenopsis orchid buds and compared with 2D skeleton-only and global 3D PCA-only baselines. The hybrid method achieved a mean angular error of 5.1° ± 3.3° (mean ± standard deviation), compared with 9.3° ± 4.2° for global 3D PCA and 14.2° ± 6.1° for the 2D skeleton method. It processed each sample in 65 ms on average and achieved a 94.0% grasp success rate (47/50) under controlled laboratory conditions.

• The method integrates 2D skeleton topology and 3D point cloud geometry in a reproducible RGB-D processing pipeline.

• Branch-wise PCA and skeleton-guided fusion improve growth-axis estimation for multi-branch orchid buds.

• The estimated growth axis is converted into a robot-referenced 6-DoF grasp pose for perception-guided manipulation.

## Graphical abstract

**Figure:**
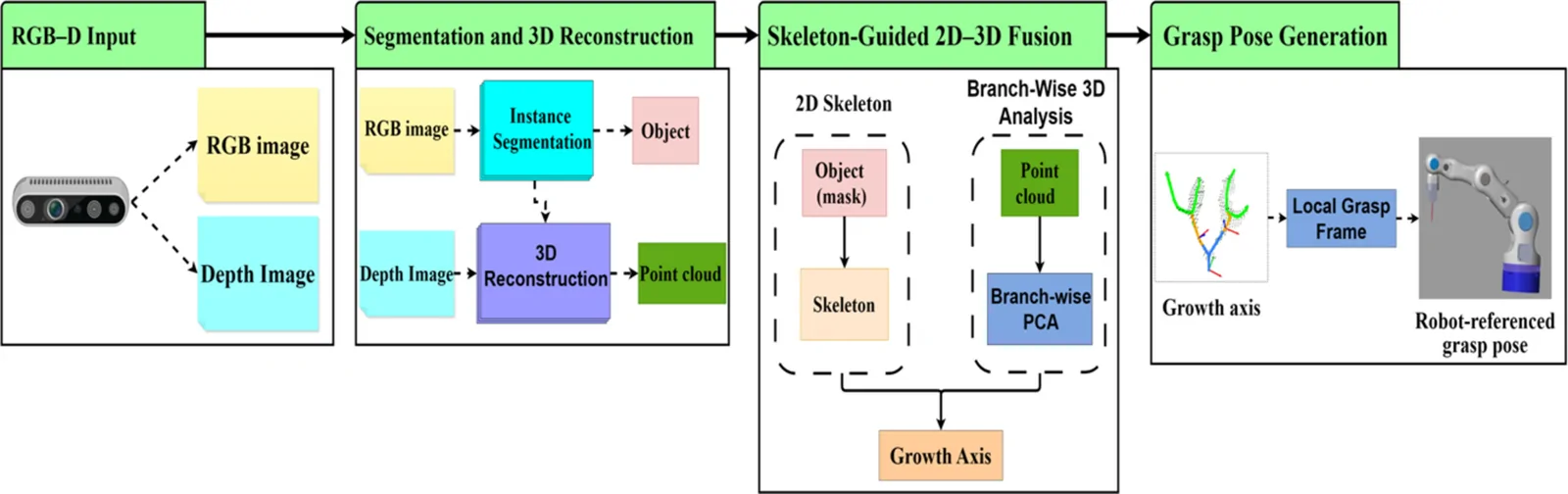

Specifications table

**Table:** 

**Subject area**	Engineering
**More specific subject area**	*RGB-D computer vision and robotic perception for plant structure analysis and agricultural manipulation*
**Name of your method**	*Hybrid 2D–3D RGB-D Growth Axis Estimation and Grasp Pose Generation Method*
**Name and reference of original method**	*Not applicable. The method is not a direct reproduction of a single previously published method. It combines and adapts established techniques, including instance segmentation, 2D skeleton extraction, RGB-D point cloud reconstruction, PCA-based orientation estimation, and skeleton-guided 2D–3D fusion, for orchid bud growth axis estimation and robotic grasp pose generation.*
Resource availability	The method was implemented in Python using OpenCV, PyTorch, Open3D, NumPy, and scikit-image. RGB-D data were acquired using an Intel RealSense D435 camera. The reproducibility package includes segmentation inference scripts, skeleton extraction scripts, point cloud reconstruction and preprocessing scripts, PCA-based axis estimation scripts, skeleton-guided fusion scripts, grasp pose generation scripts, example RGB-D inputs, calibration information, and representative output files. The source code, camera calibration files and trained segmentation weights are publicly available at https://github.com/Hieuthichcode/automated-orchid-propagation-system. The complete original dataset of 3,104 RGB images, polygon annotations, RGB-D evaluation data, and camera calibration files are available at https://drive.google.com/drive/folders/1FqTuIMWtxHYL037exPB-O3m-0CBoGs5N?usp=sharing.

## Background

Phalaenopsis orchids, commonly known as moth orchids, are commercially important ornamental plants with high economic value. The demand for high-quality orchid production has encouraged high-throughput plant phenotyping and sustainable phytoprotection technologies [1,2]. Because orchid tissues are delicate and morphologically variable, propagation and handling often require precise, customized operations [3]. In commercial production, structural analysis of orchid seedlings and buds is important for quality control, sorting, and manipulation, and 3D point-cloud-based segmentation has supported automated orchid seedling analysis [4]. However, greenhouse tasks such as bud separation, sorting, and precision handling still depend largely on manual labor, creating a need for robotic perception methods for autonomous agri-food manipulation [5].

Reliable robotic manipulation requires visual perception methods that can detect target plant organs, estimate their geometry, and provide grasp-relevant information. Object detection and segmentation models are widely used to localize targets in cluttered scenes [6]. For robotic harvesting and plant handling, 3D shape understanding is important because plant organs are often partially occluded, deformable, and surrounded by other structures [7]. Accurate pose estimation and adaptable grasp configuration are also needed to avoid damaging delicate tissues [8]. Therefore, 3D vision, machine learning, and point-cloud-based growth analysis have become useful tools for plant trait measurement, phenotyping, and robotic perception [[9], [10], [11]].

Two-dimensional image analysis provides an efficient way to extract structural information from plant organs. Instance segmentation can separate target organs from cluttered backgrounds and has been applied to plant architectural trait measurement [12]. Deep learning models such as Mask R-CNN also support instance-level object segmentation [13]. After segmentation, skeletonization can convert binary plant masks into simplified centerline structures [14]. These skeletons are useful for identifying endpoints, junctions, and branch connectivity [15]. However, 2D representations lack depth information and cannot directly describe the true spatial orientation of a bud or provide a complete geometric basis for robotic grasp pose generation [16,17].

Three-dimensional point cloud processing provides complementary geometric information. Open3D supports practical 3D data processing workflows [18], and point-cloud-based phenotyping has been applied to crops and plant structures [19]. In robotic grasping, point cloud geometry is used to estimate object shape and support grasp pose generation [20]. For plant structural analysis, 3D phenotyping pipelines have used geometric models to describe stems, branches, and internode-level structures [21]. PCA is commonly used to estimate the dominant direction of a point distribution, but global PCA can be biased by secondary branches or asymmetric point distributions in multi-branch orchid buds, reducing the reliability of downstream grasp pose generation [22].

These limitations indicate that neither 2D topology nor 3D geometry alone is sufficient for robust growth axis estimation in complex orchid buds. Therefore, this article describes a hybrid 2D–3D RGB-D method that combines skeleton-based topological cues with point-cloud-based geometric cues. In the proposed method, 2D skeleton paths describe branch organization, while branch-wise 3D PCA provides local geometric direction candidates. These two sources of information are fused to estimate the dominant growth axis and construct a grasp-oriented coordinate frame for robotic manipulation.

### Method details

The proposed method was developed to estimate the primary growth axis of Phalaenopsis orchid buds from RGB-D data and to convert the estimated axis into a robot-referenced grasp pose. This task is relevant to perception-guided manipulation, where the robot must identify a delicate target organ, estimate its spatial orientation, and define a feasible manipulation pose before contact. Recent reviews on robotic grasping have shown that reliable visual perception is a key requirement for grasp generation in complex environments [23]. In agricultural and biological applications, deep learning and image-based analysis have also been widely used for crop monitoring, structural measurement, and plant phenotyping [[24], [25], [26]].

The input data consist of synchronized RGB images and aligned depth maps acquired from an RGB-D camera. Each orchid bud is first detected and isolated using an instance segmentation model. The predicted mask defines the region of interest for both 2D skeleton extraction and mask-constrained 3D point cloud reconstruction. This step follows the general idea of using AI-assisted segmentation to isolate biological or plant structures before quantitative analysis [27]. In the present method, object detection and instance segmentation concepts are used to obtain bud-level masks from the RGB image before any 2D or 3D structural processing [28,29].

After segmentation, each binary bud mask is converted into a 2D skeleton. The skeleton preserves the visible branch connectivity of the bud and allows endpoints, junctions, and candidate branch paths to be identified. This information is useful for describing the branch organization in the image plane. However, because the skeleton is extracted from a 2D mask, it does not directly provide the true spatial direction of the bud. Therefore, the aligned depth map is used to reconstruct a 3D point cloud for each segmented bud instance. Point-cloud-based plant measurement has been used in previous structural analysis workflows [30], and here it is adapted to provide the 3D geometric information required for growth-axis estimation.

The reconstructed point cloud is then preprocessed by depth-based back-projection, statistical outlier removal, voxel downsampling, background plane removal, and coordinate normalization. These operations reduce sensor noise, remove irrelevant background structures, and provide a more consistent 3D representation for orientation estimation. PCA is first used to obtain geometric direction information from the point cloud. However, instead of relying only on global PCA of the whole bud, the proposed method applies PCA at the branch level. Branch-wise PCA provides local 3D direction candidates associated with skeleton-derived branch regions.

The final growth axis is determined by combining the 2D skeleton topology and the branch-wise 3D PCA directions. The skeleton provides branch-level structural cues, while the point cloud provides spatial geometric cues. This fusion reduces the possibility that the estimated axis is dominated by secondary branches or asymmetric point distributions. The final normalized growth axis is then used to construct a local grasp-oriented coordinate frame. Through camera–robot calibration, this local frame is transformed into the robot base coordinate system, producing a 6-DoF grasp pose for downstream robotic manipulation.

**Fig. 1:**
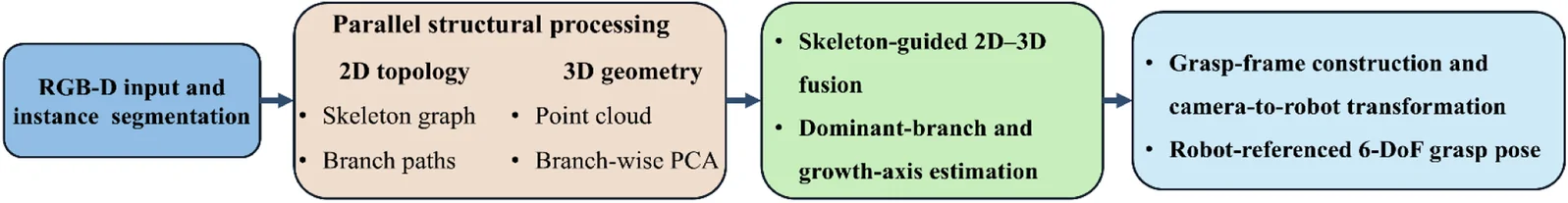
Simplified technical workflow of the proposed hybrid 2D–3D RGB-D method. After RGB-D input and instance segmentation, the segmented orchid bud is processed through parallel 2D topological and 3D geometric streams. The extracted structural and geometric cues are then fused to determine the dominant branch and estimate the growth axis, which is subsequently converted into a robot-referenced 6-DoF grasp pose.

The simplified technical workflow of the proposed method is shown in Fig. 1.

### Instance segmentation

Instance segmentation was used as the first processing stage to detect and isolate individual Phalaenopsis orchid buds from the input RGB image. The objective of this step was to generate one binary mask for each visible bud instance, so that subsequent 2D skeleton extraction and 3D point cloud reconstruction could be restricted to the target bud region. By separating each bud from the background and neighboring plant structures, this step reduces interference in the later structural and geometric analysis.

The input to this stage is an RGB image acquired from the RGB-D camera. Let the input image be denoted as:

$$I_{\text{RGB}}\in R^{H\times W\times 3}$$

whereHandWare the image height and width, respectively. The segmentation model maps$\;I_{\text{RGB}}\;$to a set of binary instance masks:

(1) $$\begin{array}{cccc} & M={\left\{M_{k}\right\}}_{k=1}^{N} & & \end{array}$$

**Fig. 2:**
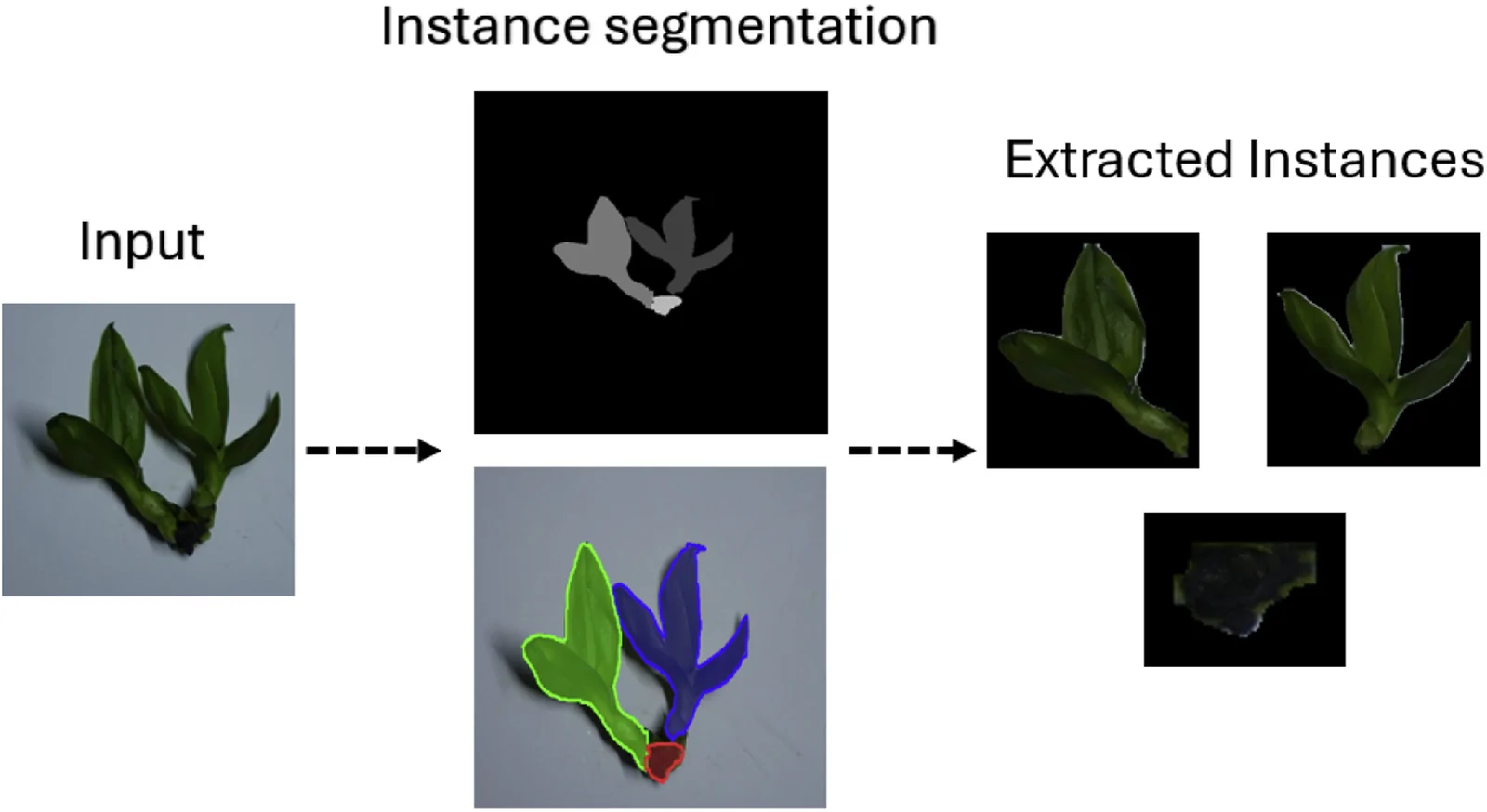
Instance segmentation results for Phalaenopsis orchid buds. From left to right: input RGB image, predicted instance masks, and separated bud instances used for 2D skeleton extraction and mask-constrained 3D point cloud reconstruction.

**Fig. 3:**
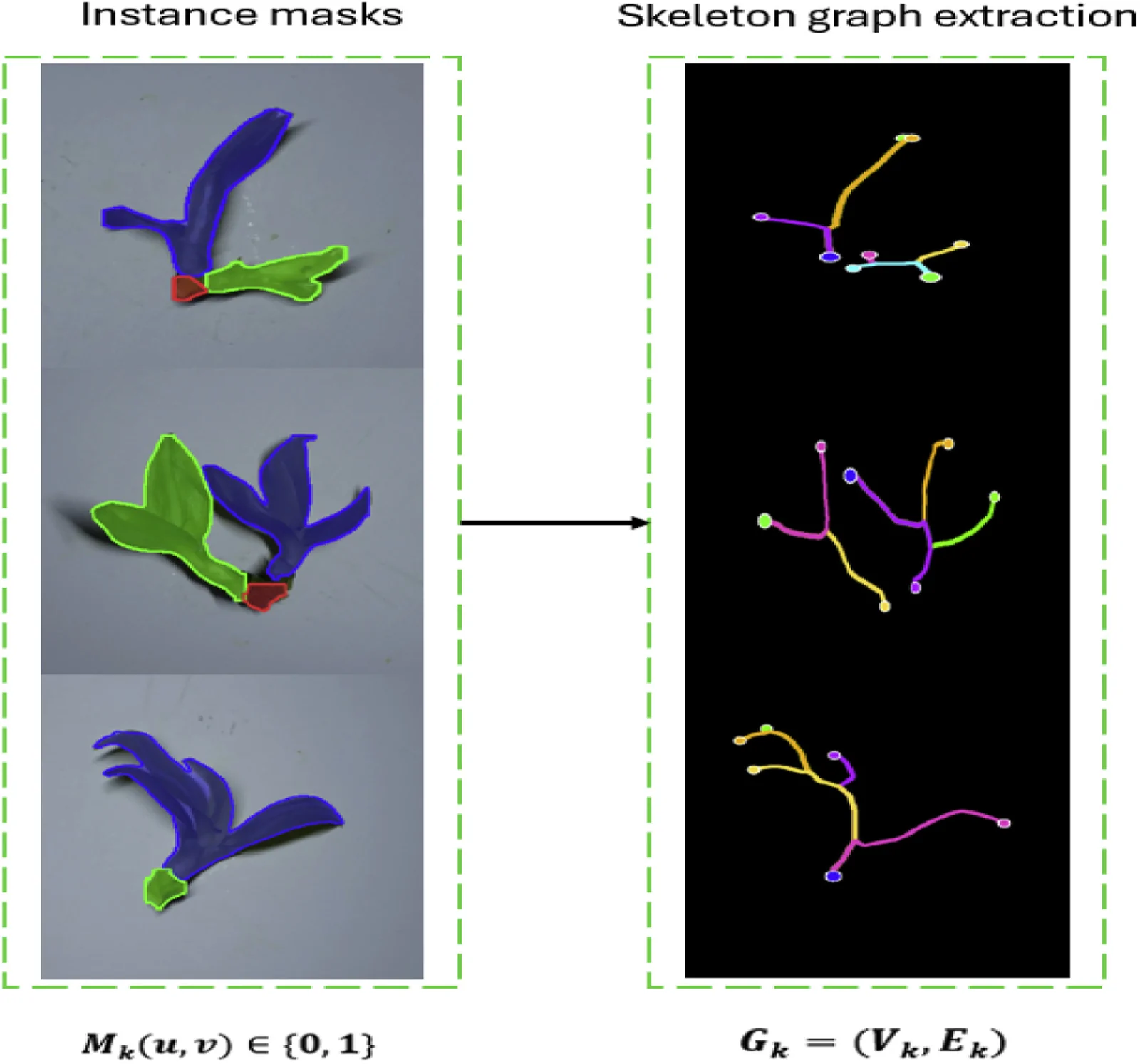
Examples of 2D skeleton graph extraction from segmented Phalaenopsis orchid bud masks. Each refined binary mask$\;{\tilde{M}}_{k}\;$is transformed into a skeleton$\;S_{k}\;$and then represented as a graph$\;G_{k}=\left(V_{k},E_{k}\right)$, where nodes denote skeleton pixels and edges represent eight-neighborhood connectivity between adjacent skeleton pixels. Endpoints and junction points are identified from the node degree and used to define candidate branch paths.

**Fig. 4:**
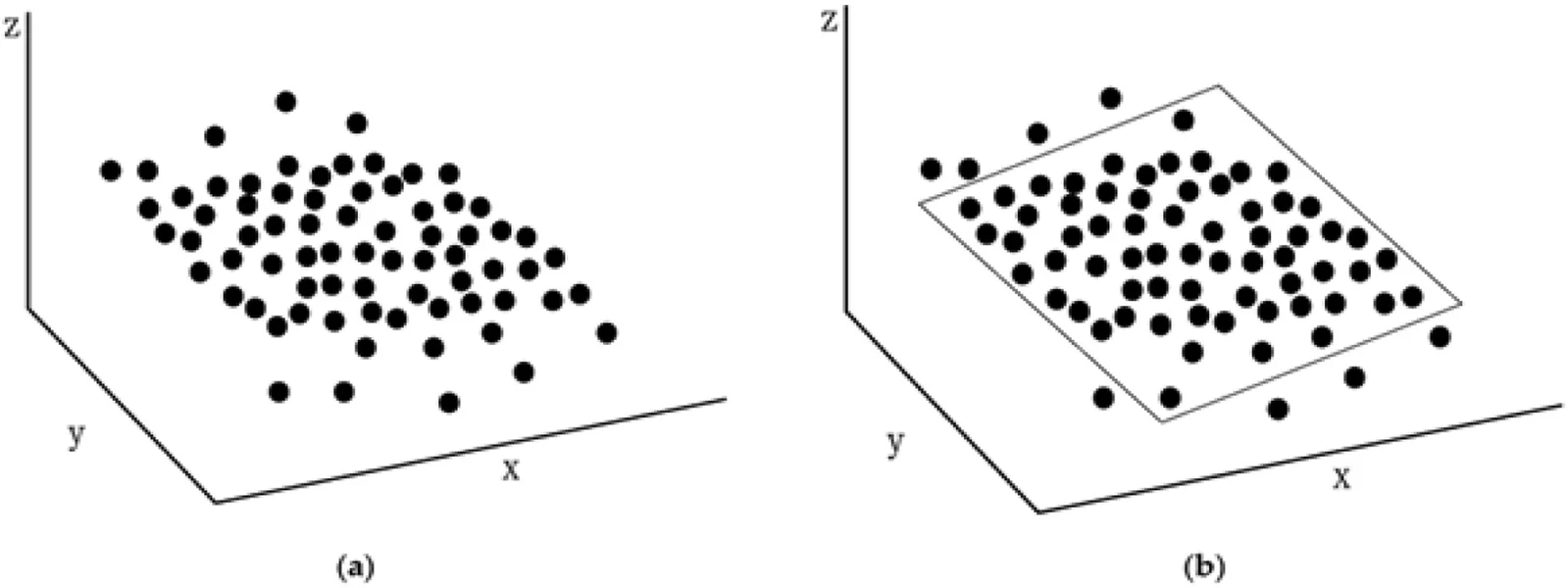
Illustration of background plane estimation using the RANSAC algorithm. (a) Original point cloud containing both inliers and outliers. (b) The dominant background plane detected from the point cloud.

**Fig. 5:**
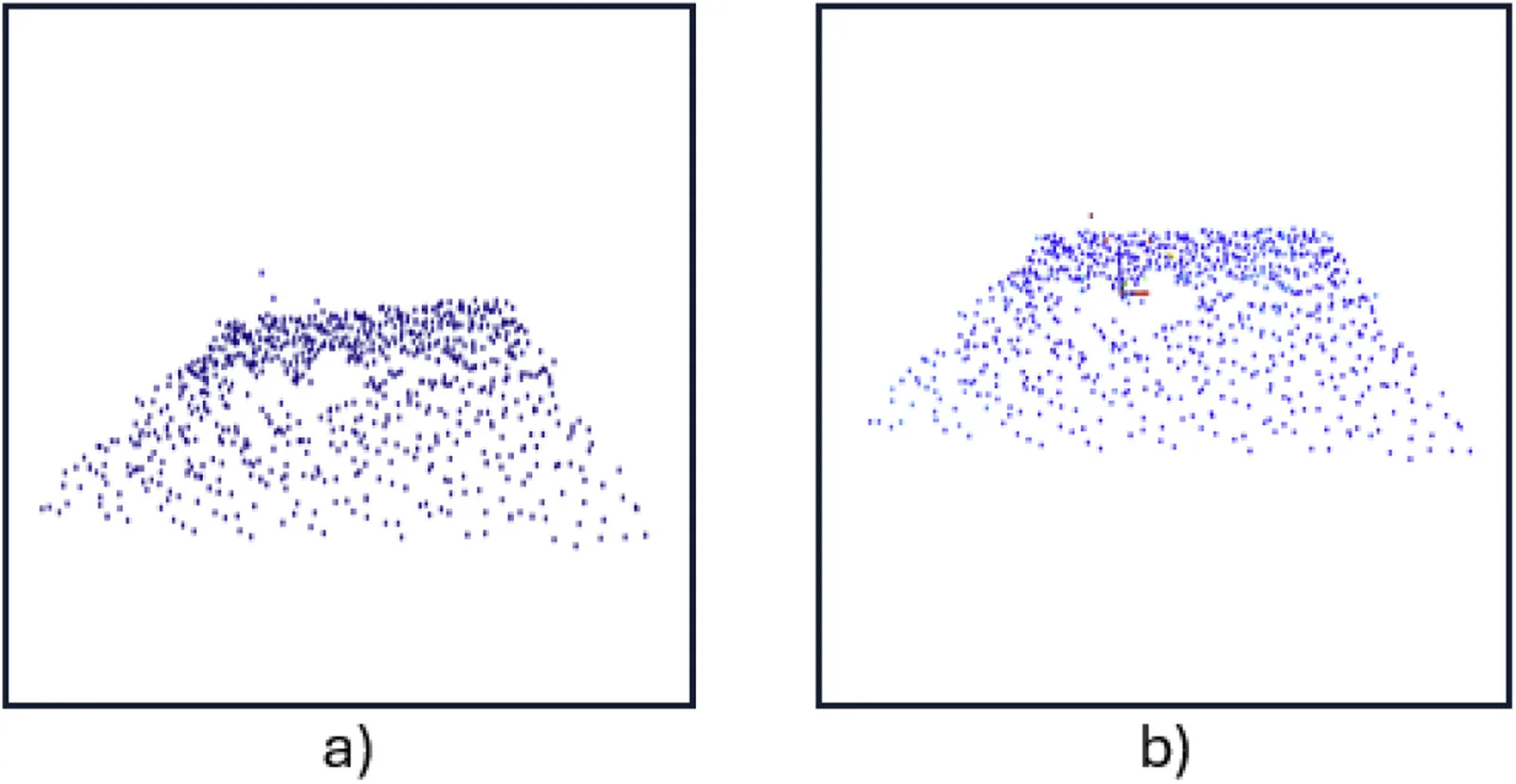
Point cloud preprocessing after RANSAC-based background removal. (a) Reconstructed point cloud before removing the detected supporting plane. (b) Processed point cloud after plane inlier removal, retaining the main orchid bud structure for subsequent PCA-based orientation estimation.

**Fig. 6:**
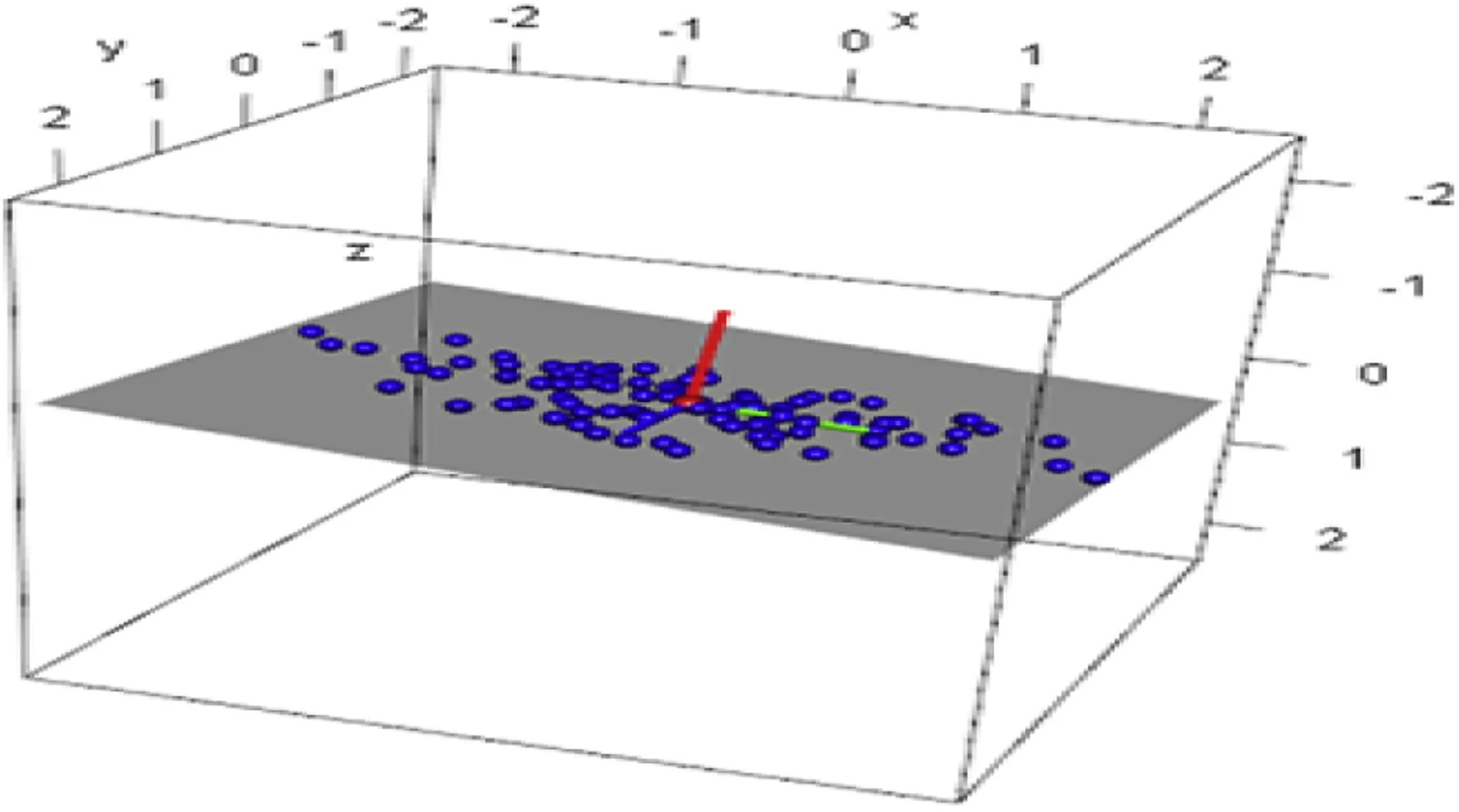
Global PCA-based dominant direction estimation on a normalized orchid bud point cloud. The principal eigenvector associated with the largest eigenvalue of the covariance matrix represents the main geometric trend of the whole point distribution. This direction is used as an initial geometric reference rather than the final growth axis.

**Fig. 7:**
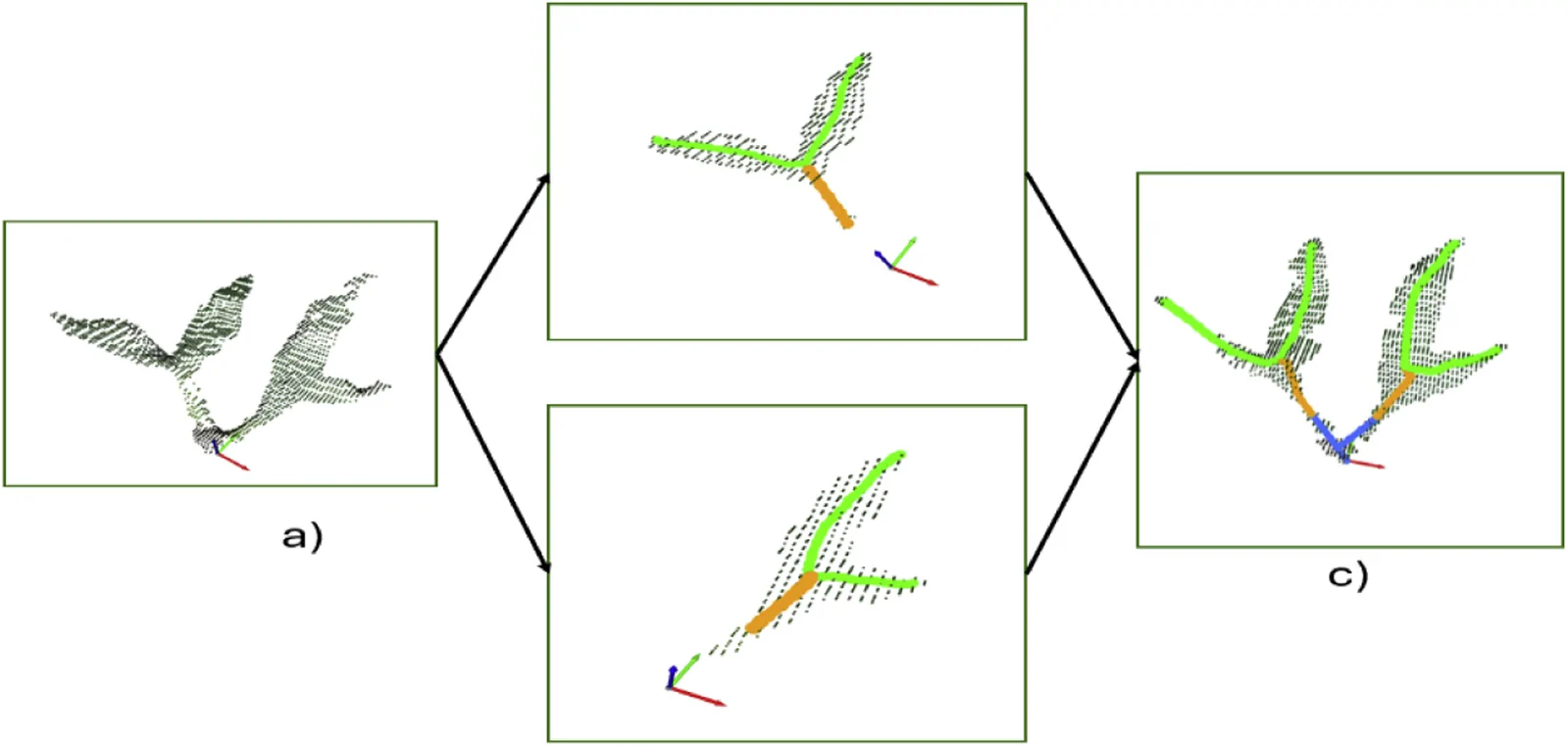
Branch-wise PCA axis estimation for an orchid bud point cloud. (a) Preprocessed point cloud of the segmented bud. (b) Candidate branch regions defined from the 2D skeleton paths and mapped into 3D space. (c) Local PCA directions estimated from the branch-specific point clouds and used as geometric candidates for skeleton-guided growth axis determination.

**Fig. 8:**
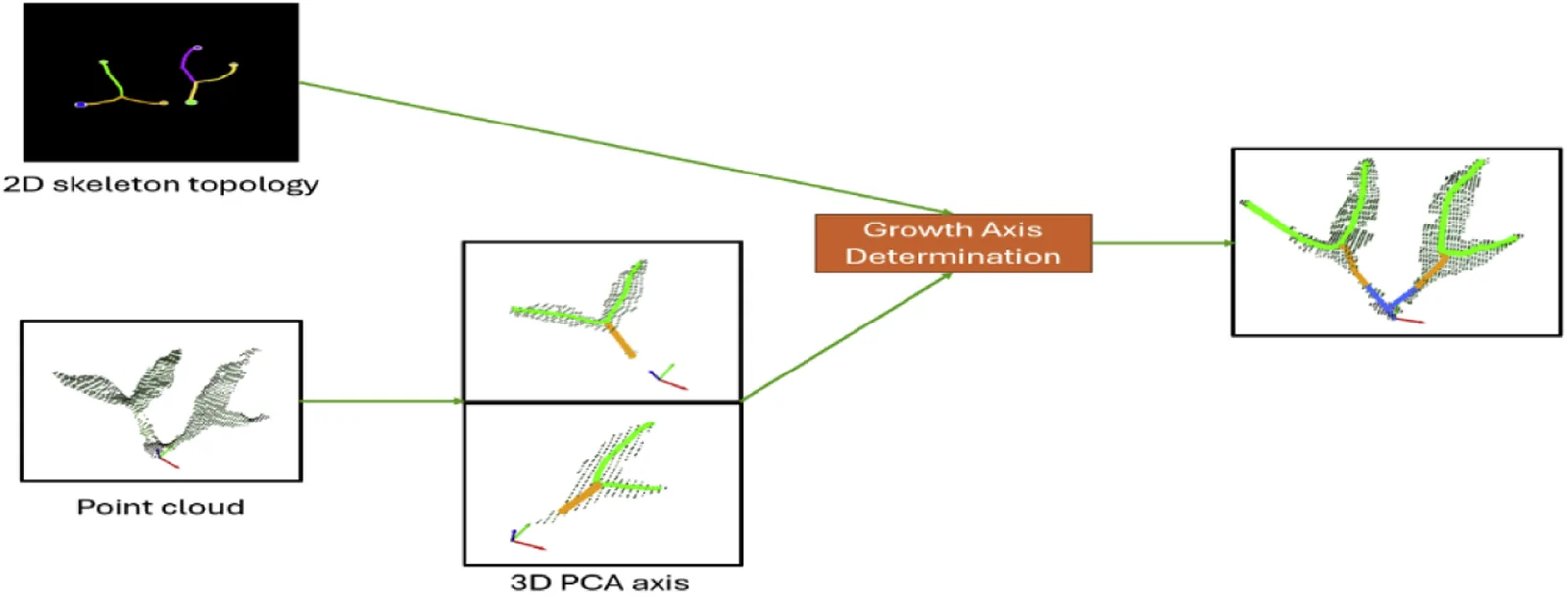
Growth axis determination using skeleton-guided 2D–3D fusion. Candidate branch paths are extracted from the 2D skeleton graph and mapped to 3D space. For each candidate branch, the skeleton-derived direction is fused with the local PCA-based direction. The selected branch provides the final normalized growth axis for grasp pose generation.

whereNis the number of detected orchid bud instances and$\;M_{k}\in {\left\{0,1\right\}}^{H\times W}\;$is the binary mask associated with thek-th bud instance. Each mask identifies the pixel region of one orchid bud and allows that bud to be processed independently from other objects in the scene. Fig. 2,3,4,5,6,7,8

In this implementation, a YOLOv8s-seg instance-segmentation model was trained using a dataset of Phalaenopsis orchid bud images prepared and annotated on the Roboflow platform. The original dataset comprised 3,104 RGB images. Each visible orchid bud was manually annotated as an individual instance using a polygon-based segmentation mask.

Before any stochastic data augmentation was applied, the 3,104 original images were partitioned into training, validation, and test subsets at a ratio of 75:15:10. This partitioning was performed at the original-image level to prevent augmented versions of the same source image from appearing in different subsets. Data augmentation was subsequently applied only to the training subset, whereas the validation and test subsets were not augmented.

Deterministic preprocessing included automatic orientation correction, contrast adjustment, reflected-edge padding, and resizing to 1024 × 1024 pixels while preserving the original image aspect ratio. For the training subset only, the augmentation operations included horizontal and vertical flipping; 90° rotations; random rotations between −12° and +12°; horizontal and vertical shearing of up to ±15° and ±14°, respectively; saturation adjustment between −26% and +26%; brightness adjustment between −6% and +6%; Gaussian blur of up to 0.2 pixels; and noise addition affecting up to 0.02% of image pixels. The corresponding polygon annotations were transformed consistently with each augmented image. After preprocessing and augmentation, the final training set contained five images derived from each original training image, yielding 11,640 training images from the 2,328 original training images. The complete dataset therefore contained 12,416 images: 11,640 training images, 466 validation images, and 310 test images.

The model was trained for 380 epochs using the AdamW optimizer with a learning rate of 0.001. The resulting segmentation model achieved a mask mAP@0.5 of 0.970 on the validation set, indicating that the predicted instance masks were sufficiently reliable for the subsequent skeleton extraction and point-cloud reconstruction stages.

After mask prediction, simple morphological post-processing was applied to improve mask quality. Erosion and dilation were used to suppress uncertain boundary pixels, remove small isolated artifacts, and fill minor holes inside the segmented bud regions. The refined mask of thek-th bud is denoted as:

(2) $${\tilde{M}}_{k}=\Phi \left(M_{k}\right)$$

whereΦ(·)represents the morphological refinement operation. In this study, the morphological kernel size was set to 3 × 3 and the number of erosion/dilation iterations was set to 2.

The refined masks were then used in two ways. First, each$\;{\tilde{M}}_{k}\;$was used to extract the corresponding RGB bud instance for visual inspection and 2D structural analysis. Second, the same mask was used to constrain the valid pixel region for depth back-projection, so that only pixels belonging to the segmented bud were reconstructed into 3D space. This mask-constrained reconstruction substantially reduces background noise in the generated point cloud and improves the reliability of later PCA-based orientation estimation.

Because the proposed method depends on both 2D topology and 3D geometry, segmentation quality directly affects the reliability of subsequent processing stages. Therefore, instance segmentation serves not only as an object detection step, but also as the basis for constructing a consistent orchid bud representation across the RGB image, 2D skeleton graph, and 3D point cloud domains.

### Skeleton extraction (2D)

After obtaining the segmented orchid bud instances, each refined binary mask was converted into a 2D skeleton representation to analyze the structural topology of the bud. The purpose of this step was to reduce the segmented bud region to a compact centerline structure while preserving its branch connectivity. This representation provides topological cues for identifying endpoints, junction points, and candidate growth branches.

Let the refined binary mask of thek-th bud instance be denoted as:

(3) $${\tilde{M}}_{k}\in {\left\{0,1\right\}}^{H\times W}$$

Skeletonization was applied to$\;{\tilde{M}}_{k}\;$to obtain a one-pixel-wide skeleton:

(4) $$S_{k}=\Psi \left({\tilde{M}}_{k}\right)$$

whereΨ(·)denotes the skeletonization operation. In this study, a thinning-based skeletonization algorithm was used to preserve the connectivity of the segmented bud region while reducing it to a medial-axis-like representation.

To enable structural analysis, the skeleton was represented as an undirected graph:

(5) $$G_{k}=\left(V_{k},E_{k}\right)$$

where$\;V_{k}\;$is the set of skeleton pixels treated as graph nodes, and$\;E_{k}\;$is the set of edges connecting neighboring skeleton pixels. Eight-neighborhood connectivity was used to construct the graph, so two skeleton pixels were connected if they were adjacent horizontally, vertically, or diagonally.

For each node$\;v_{i}\in V_{k}$, the node degree was computed as:

(6) $$\text{deg}\left(v_{i}\right)=|\left\{v_{j}\in V_{k}|\left(v_{i},v_{j}\right)\in E_{k}\right\}|$$

Nodes with degree one were defined as endpoints:

(7) $$V_{\text{end}}=\left\{v_{i}\in V_{k}|\text{deg}\left(v_{i}\right)=1\right\}$$

whereas nodes with degree greater than two were defined as junction points:

(8) $$V_{\text{junc}}=\left\{v_{i}\in V_{k}|\text{deg}\left(v_{i}\right)>2\right\}$$

The endpoints generally correspond to visible bud tips or branch tips, while the junction points indicate branching regions. These structural features are used to identify candidate branch paths in the skeleton graph.

To define the branch direction, a basal node was first selected from the skeleton. In the present implementation, the basal node$\;b_{k}\;$was selected as the skeleton endpoint located closest to the lower region of the segmented mask. If several candidate endpoints were located near the lower boundary, the endpoint closest to the centroid of the lower mask region was selected. Each candidate branch path was then defined as the shortest path in the skeleton graph from the basal node$\;b_{k}\;$to one endpoint$\;e_{m}\in V_{\text{end}}$:

(9) $$\pi_{m}=\text{path}\left(b_{k},e_{m}\right)$$

where$\;\pi_{m}\;$denotes them-th candidate branch path. These paths provide a graph-based description of the visible branch organization of the orchid bud.

Although the skeleton is extracted only in the 2D image domain, it provides useful topological information that cannot be obtained from point cloud covariance alone. In particular, for multi-branch orchid buds, the skeleton graph helps distinguish the dominant branch path from secondary branches. Therefore, the extracted skeleton is used as an intermediate structural representation that later guides the fusion with 3D geometric information.

The output of this stage is a skeleton graph$\;G_{k}$, a set of endpoints$\;V_{\text{end}}$, a set of junction points$\;V_{\text{junc}}$, the basal node$\;b_{k}$, and a set of candidate branch paths$\;\{\pi_{m}\}\;$for each segmented bud instance.

### Point Cloud Reconstruction and Processing

After instance segmentation and 2D skeleton extraction, the next step was to reconstruct the 3D geometry of each orchid bud from the RGB-D data. The purpose of this stage was to generate a clean and spatially consistent point cloud for each segmented bud instance while reducing the influence of sensor noise, missing depth values, and background structures.

Two coordinate frames are used in the following 3D processing stages. The camera coordinate frame, denoted by{C}, is the native frame of the RGB-D camera in which the points are initially reconstructed. The plane-normalized coordinate frame, denoted by{N}, is obtained by rotating the camera-frame point cloud so that the normal of the detected supporting plane is aligned with the positivez-axis of{N}.

For each bud instance, only pixels belonging to the refined foreground mask$\;{\tilde{M}}_{k}\;$were used for depth back-projection of the bud-specific point cloud. LetD(u,v)denote the depth value at image coordinate(u,v), whereuandvare the horizontal and vertical pixel coordinates, respectively. The intrinsic camera matrix of the RGB-D camera is defined as:

(10) $$K=\left[\begin{array}{ccc} f_{x} & 0 & c_{x}\\ 0 & f_{y} & c_{y}\\ 0 & 0 & 1 \end{array}\right]$$

where$\;f_{x}\;$and$\;f_{y}\;$are the focal lengths, and$\;c_{x}\;$and$\;c_{y}\;$are the principal point coordinates. For each valid foreground pixel satisfying$\;{\tilde{M}}_{k}\left(u,v\right)=1\;$andD(u,v)>0, the corresponding 3D point in the camera coordinate system was reconstructed using the pinhole camera model:

(11) $$z_{i}^{C}=D\left(u_{i},v_{i}\right)$$

(12) $$\begin{array}{cccc} & x_{i}^{C}=\frac{\left(u_{i}-c_{x}\right)z_{i}^{C}}{f_{x}} & & \end{array}$$

(13) $$\begin{array}{cccc} & y_{i}^{C}=\frac{\left(v_{i}-c_{y}\right)z_{i}^{C}}{f_{y}} & & \end{array}$$

Thus, the reconstructed point cloud of thek-th orchid bud instance is:

(14) $$P_{k}^{C}={\left\{p_{i}^{C}={\left[x_{i}^{C},y_{i}^{C},z_{i}^{C}\right]}^{T}|{\tilde{M}}_{k}\left(u_{i},v_{i}\right)=1,|D\left(u_{i},v_{i}\right)>0\right\}}_{i=1}^{N_{k}}$$

where$\;N_{k}\;$is the number of valid 3D points reconstructed for thek-th bud. Because the reconstruction was restricted to the segmented foreground region, irrelevant points from surrounding plant parts and background regions were reduced at the earliest stage of 3D processing. Concurrently, a local region of interest surrounding each segmented bud was retained to provide scene points for RANSAC-based supporting-plane estimation.

However, point clouds reconstructed from RGB-D data may still contain isolated noisy points, non-uniform point density, missing depth regions, and residual background surfaces. Therefore, several preprocessing operations were applied before orientation estimation. First, statistical outlier removal was used to remove isolated points whose local neighborhoods deviated from the main point distribution. For each point, the mean distance to its nearest neighbors was computed, and points with mean distances larger than a standard deviation threshold were removed. In this study, the number of nearest neighbors was set to 10 and the standard deviation ratio was set to 2.

Second, voxel downsampling was applied to reduce point density while preserving the overall geometry of the bud. This step reduces computational cost and improves numerical stability in the following PCA-based orientation estimation. The voxel size was set to 0.5 mm in this study.

After initial denoising and downsampling, the dominant supporting plane was estimated by applying RANSAC to the local region of interest, while the refined foreground mask was retained to preserve the bud-specific point cloud. The RANSAC plane model and the associated inlier test were evaluated in the camera coordinate frame{C}. The plane model is expressed as:

(15) $$ax^{C}+by^{C}+cz^{C}+d=0$$

or equivalently:

(16) $${\left(n_{p}^{C}\right)}^{T}p_{i}^{C}+d=0$$

where$\;n_{p}^{C}={\left[a,b,c\right]}^{T}\;$is the unit plane-normal vector expressed in the camera coordinate frame{C}, anddis the correspondingly normalized plane offset, such that$\;∥n_{p}^{C}∥=1$. A point$\;p_{i}^{C}\;$was classified as a plane inlier if:

(17) $$|{\left(n_{p}^{C}\right)}^{T}p_{i}^{C}+d|<\tau_{p}$$

where$\;\tau_{p}\;$is the point-to-plane distance threshold. In this implementation, the RANSAC distance threshold was set to 3 mm, and the maximum number of iterations was set to 500. The estimated plane model was then used to remove residual supporting-plane points from the mask-constrained bud point cloud.

Using the coordinate frames defined above, the filtered camera-frame point cloud was rotated to reduce viewpoint-dependent variation. Let$\;n_{p}^{C}\;$denote the unit normal vector of the detected plane expressed in the camera frame and let$\;e_{z}^{N}={\left[0,0,1\right]}^{T}\;$denote the positivez-axis of the plane-normalized frame. A rotation matrix$\;R_{C}^{N}\;$was computed such that:

(18) $$\begin{array}{cccc} & R_{C}^{N}n_{p}^{C}=e_{z}^{N} & & \end{array}$$

The matrix$\;R_{C}^{N}\;$maps points and vectors from the camera frame{C}to the plane-normalized frame{N}. Each filtered point was transformed as:

(19) $$p_{i}^{N}=R_{C}^{N}p_{i}^{C}$$

Because only rotational normalization was applied, no translational offset was introduced between the two frames. The normalized point cloud of thek-th bud was therefore:

(20) $$\begin{array}{cccc} & P_{k}^{N}={\left\{p_{i}^{N}\right\}}_{i=1}^{{\tilde{N}}_{k}} & & \end{array}$$

where$\;{\tilde{N}}_{k}\;$is the number of points remaining after statistical outlier removal, voxel downsampling, and RANSAC-based plane removal.

Since$\;R_{C}^{N}\;$is a rotation matrix, the inverse transformation is$\;R_{N}^{C}={\left(R_{C}^{N}\right)}^{T}\;$. All subsequent global PCA, branch-wise PCA, and skeleton-guided directional fusion operations were performed in{N}. Skeleton points initially deprojected in{C}were transformed using the same matrix$\;R_{C}^{N}\;$before fusion. The final estimated axis was transformed back to the camera frame using$\;d^{C}=R_{N}^{C}d^{N}\;$before camera-referenced angular evaluation and robotic grasp-pose generation.

The output of this stage is a denoised, downsampled, background-filtered point cloud expressed in the plane-normalized frame, together with the forward and inverse rotations required to maintain coordinate-frame consistency throughout the subsequent processing stages.

### PCA-based axis estimation

After point cloud reconstruction and preprocessing, Principal Component Analysis (PCA) was applied to estimate the dominant geometric orientation of each orchid bud in 3D space. The purpose of this stage was to obtain one or more candidate direction vectors that describe the main spatial trend of the reconstructed point cloud. These PCA-based directions were used as geometric candidates and were later refined using the 2D skeleton topology.

Let the plane-normalized point cloud of thek-th orchid bud instance be defined as:

(21) $$P_{k}^{N}={\left\{p_{i}^{N}\right\}}_{i=1}^{{\tilde{N}}_{k}},\;p_{i}^{N}\in R^{3}$$

where$\;{\tilde{N}}_{k}\;$is the number of points in the preprocessed point cloud. The centroid of the point cloud was first computed as:

(22) $${\bar{P}}_{k}^{N}=\frac{1}{\tilde{N_{k}}}\sum_{i=1}^{{\tilde{N}}_{k}}P_{i}^{N}$$

The covariance matrix describing the spatial distribution of the plane-normalized point cloud was then calculated as:

(23) $$\Sigma_{k}^{N}=\frac{1}{{\tilde{N}}_{k}}\sum_{i=1}^{{\tilde{N}}_{k}}\left(p_{i}^{N}-p_{k}^{-N}\right){\left(p_{i}^{N}-p_{k}^{-N}\right)}^{T}$$

Eigenvalue decomposition was performed on the covariance matrix:

(24) $$\Sigma_{k}^{N}q_{j}^{N}=\lambda_{j}q_{j}^{N},\;j\in \left\{1,\;2,\;3\right\}$$

where$\;\lambda_{j}\;$is thej-th eigenvalue and$\;q_{j}^{N}\;$is the corresponding eigenvector expressed in the plane-normalized frame{N}. The eigenvector associated with the largest eigenvalue was selected as the PCA-based dominant direction:

(25) $$d_{\text{PCA}}^{N}=q_{\text{max}}^{N},\;q_{\text{max}}^{N}=q_{j}^{N}\;\text{where}\;\lambda_{j}=\text{max}\left(\lambda_{1},\lambda_{2},\lambda_{3}\right)$$

This vector represents the main geometric direction of the point distribution in the plane-normalized frame{N}. However, for multi-branch orchid buds, the global PCA direction can be biased by secondary branches or asymmetric point distributions. Therefore, the global PCA result was used only as an initial geometric reference and not as the final growth axis.

To improve robustness, PCA was also applied at the branch level. Candidate branch paths$\;\{\pi_{m}\}$, extracted from the 2D skeleton graph, were used to define structurally meaningful branch regions. For each branch path$\;\pi_{m}$, pixels located within a distance threshold$\;r_{s}\;$from the skeleton path were assigned to the corresponding branch region. Let$\;{\tilde{P}}_{k}^{N}$denote the pre-downsampled plane-normalized point cloud of thek-th bud, with the original image coordinate associated with each point retained. The corresponding 3D points were then selected from$\;{\tilde{P}}_{k}^{N}\;$to form a pre-downsampled branch-specific point cloud:

(26) $${\tilde{P}}_{k,m}^{N}=\left\{{\tilde{p}}_{i}^{N}\in {\tilde{P}}_{k}^{N}|\text{dist}\left(\left(u_{i},v_{i}\right),\pi_{m}\right)<r_{s}\right\}$$

where$\;{\tilde{P}}_{k,m}^{N}\;$is the pre-downsampled point cloud associated with them-th candidate branch of thek-th bud,$\;(u_{i},v_{i})\;$is the original image coordinate associated with the pre-downsampled 3D point$\;{\tilde{p}}_{i}^{N}$, and$\;r_{s}\;$is the pixel-distance threshold around the skeleton path. Each resulting branch-specific point cloud$\;{\tilde{P}}_{k,m}^{N}\;$was subsequently voxel-downsampled to obtain$\;P_{k,m}^{N}\;$before branch-wise PCA.

In this study,$\;r_{s}\;$was set to 5 pixels. The selection of$\;r_{s}=5\;$pixels is quantitatively supported by the sensitivity analysis presented in Table 1. Accordingly, a 3D point was assigned to them-th branch when its corresponding image pixel was located at a Euclidean pixel distance of less than 5 pixels from the skeleton path$\;\pi_{m}$. This neighborhood was selected to retain a sufficient number of valid depth points for stable branch-wise PCA estimation while limiting the inclusion of points belonging to adjacent branches. When a pixel fell within the support regions of more than one candidate branch, it was assigned to the nearest skeleton path according to the minimum Euclidean pixel distance.

**Table 1:** Sensitivity of growth-axis estimation to the skeleton neighborhood threshold$\;r_{s}$.

$r_{s}\;$**(pixels)**	**Mean angular error (°)**	**Standard deviation (°)**	**Valid branch rate (%)**	**Overlapping support rate (%)**
2	7.8	4.6	91.0	0.5
3	6.6	4.1	95.4	1.0
4	5.8	3.7	98.0	1.8
5	5.3	3.4	99.0	3.0
6	5.4	3.5	99.2	4.8
7	5.7	3.7	99.4	7.2
8	6.0	4.0	99.6	10.2
9	6.4	4.3	99.6	13.8
10	6.9	4.7	99.8	18.1
11	7.5	5.1	99.8	23.0

PCA was then applied separately to each branch-specific point cloud$\;P_{k,m}^{N}$. For each branch, the centroid and covariance matrix were first computed from the points in$\;P_{k,m}^{N}$. Eigenvalue decomposition was then performed on the covariance matrix. The eigenvector corresponding to the largest eigenvalue was selected as the local PCA direction of the branch:

(27) $$d_{\text{PCA},k,m}^{N}=v_{k,m,1}^{N}$$

(28) $$\lambda_{k,m,1}\geq \lambda_{k,m,2}\geq \lambda_{k,m,3}$$

where$\;d_{\text{PCA},k,m}^{N}\;$is the PCA-based direction of them-th candidate branch of thek-th bud instance expressed in the plane-normalized frame{N}, and$\;v_{k,m,1}^{N}\;$is the corresponding principal eigenvector associated with the largest eigenvalue$\;\lambda_{k,m,1}$. The output of this stage is a set of PCA-based candidate directions:

(29) $$D_{k,\;\text{PCA}}^{N}={\left\{d_{\text{PCA},k,\;m}^{N}\right\}}_{m=1}^{M_{k}}$$

where$\;M_{k}\;$is the number of candidate branch paths extracted from the 2D skeleton of thek-th bud. All candidate directions in$\;D_{k,\text{PCA}}^{N}\;$are expressed in the plane-normalized coordinate frame{N}.

### Skeleton-guided growth axis determination

Although PCA provides useful geometric direction candidates from the reconstructed 3D point cloud, the PCA-based direction alone may still be affected by secondary branches or locally unbalanced point distributions. Therefore, the final growth axis was not determined from point cloud geometry alone. Instead, this step combines branch-wise PCA direction candidates with structural cues extracted from the 2D skeleton graph. The purpose of this stage is to select the dominant branch and estimate one normalized growth axis for each segmented orchid bud instance.

For each segmented bud instance, the skeleton graph$\;G_{k}=\left(V_{k},E_{k}\right)$, the basal node$\;b_{k}$, and the candidate branch paths$\;{\left\{\pi_{m}\right\}}_{m=1}^{M_{k}}\;$were obtained from the 2D skeleton extraction stage. Each candidate path$\;\pi_{m}\;$connects the basal node$\;b_{k}\;$to an endpoint$\;e_{m}$, and therefore represents one possible growth branch in the image domain. The corresponding 3D branch direction was estimated by mapping the basal node and endpoint of each path to 3D coordinates using the aligned depth map and transforming the resulting points from the camera frame{C}to the plane-normalized frame{N}using$\;R_{C}^{N}$.

Let$\;p_{b,k}^{N}\;$denote the 3D point corresponding to the basal node$\;b_{k}$, and let$\;p_{e,k,m}^{N}\;$denote the 3D point corresponding to the endpoint$\;e_{m}\;$of them-th candidate path of thek-th bud, both expressed in the plane-normalized frame{N}.

(30) $$d_{\text{skel},k,m}^{N}=\frac{p_{e,k,m}^{N}-p_{b,k}^{N}}{∥p_{e,k,m}^{N}-p_{b,k}^{N}∥}$$

where$\;d_{\text{skel},k,m}^{N}\;$represents the base-to-tip direction inferred from the skeleton topology after mapping to 3D space and transformation into the plane-normalized frame{N}.

For the same branch region, branch-wise PCA provides a local geometric direction$\;d_{\text{PCA},k,m}^{N}$. Since PCA eigenvectors are sign-ambiguous, an oriented PCA direction$\;{\tilde{d}}_{\text{PCA},k,m}^{N}\;$was defined by aligning the sign of$\;d_{\text{PCA},k,m}^{N}\;$with the skeleton-derived base-to-tip direction before fusion:

(31) $${\tilde{d}}_{\text{PCA},k,m}^{N}=\left\{ \begin{array}{cc} d_{\text{PCA},k,m}^{N}, & \text{if}\;{\left(d_{\text{PCA},k,m}^{N}\right)}^{T}d_{\text{skel},k,m}^{N}\geq 0,\\ -d_{\text{PCA},k,m}^{N}, & \text{otherwise}. \end{array}\right.$$

The fused direction of them-th candidate branch of thek-th bud was then computed as a weighted combination of the sign-aligned PCA-based geometric direction and the skeleton-derived structural direction:

(32) $$d_{\text{fuse},k,m}^{N}=\alpha {\tilde{d}}_{\text{PCA},k,m}^{N}+\left(1-\alpha \right)d_{\text{skel},k,m}^{N}$$

whereα∈[0,1]is a weighting parameter that controls the relative contribution of the 3D geometric cue and the skeleton-derived structural cue. A largerαgives more importance to the PCA-based point cloud direction, whereas a smallerαgives more importance to the skeleton-derived branch direction. In this implementation,αwas set to 0.6 to slightly prioritize the PCA-based geometric direction while still retaining the contribution of the skeleton-derived structural direction. The selection and robustness ofα=0.6are quantitatively evaluated in Table 2. The fused direction was normalized as:

(33) $${\hat{d}}_{k,m}^{N}=\frac{d_{\text{fuse},k,m}^{N}}{∥d_{\text{fuse},k,m}^{N}∥}$$

**Table 2:** Sensitivity of growth-axis estimation to the fusion weightα.

α	**Overall error (mean ± SD, °)**	**Single-branch error (mean ± SD, °)**	**Multi-branch error (mean ± SD, °)**
0.0	9.0 ± 4.7	7.6 ± 4.0	10.9 ± 5.2
0.1	8.1 ± 4.4	6.9 ± 3.7	9.8 ± 4.9
0.2	7.2 ± 4.1	6.1 ± 3.4	8.7 ± 4.5
0.3	6.4 ± 3.8	5.4 ± 3.2	7.8 ± 4.2
0.4	5.8 ± 3.6	4.9 ± 3.0	7.0 ± 3.9
0.5	5.3 ± 3.4	4.5 ± 2.9	6.4 ± 3.7
0.6	5.1 ± 3.3	4.3 ± 2.8	6.2 ± 3.6
0.7	5.2 ± 3.4	4.4 ± 2.9	6.3 ± 3.7
0.8	5.7 ± 3.6	4.8 ± 3.1	6.9 ± 3.9
0.9	6.5 ± 3.9	5.5 ± 3.3	7.9 ± 4.3
1.0	7.7 ± 4.3	6.5 ± 3.6	9.3 ± 4.7

To select the final dominant growth axis, each candidate branch was evaluated based on its skeleton path length and its angular consistency with the sign-aligned local PCA direction. In the present implementation, the dominant branch was selected as the basal-to-endpoint path with the longest skeleton path length, provided that the angular difference between$\;{\tilde{d}}_{\text{PCA},k,m}^{N}\;$and$\;d_{\text{skel},k,m}^{N}\;$remained below a predefined threshold$\;\theta_{\text{max}}$. The angular difference was computed as:

(34) $$\theta_{k,m}=\frac{180}{\pi }\text{co}s^{-1}\left[{\left({\tilde{d}}_{\text{PCA},k,m}^{N}\right)}^{T}d_{\text{skel},k,m}^{N}\right]$$

where$\;\theta_{k,m}\;$is the angular difference between the sign-aligned local PCA direction and the skeleton-derived direction for them-th candidate branch of thek-th bud.

The maximum angular-difference threshold was set to$\;\theta_{\text{max}}=85^{\circ }$. This relatively permissive threshold was used to tolerate large local orientation differences caused by curved bud geometry, partial occlusion, sparse depth measurements, and local point-cloud imbalance. Candidate branches with$\;\theta_{k,m}>\theta_{\text{max}}\;$were treated as geometrically inconsistent and were not selected as the dominant branch. If no candidate branch satisfied this threshold, the longest basal-to-endpoint skeleton path was selected as a fallback. The final growth axis was then defined as:

(35) $${\hat{d}}_{k}^{N}={\hat{d}}_{k,m^{*}}^{N}$$

where$\;m^{*}\;$denotes the selected dominant branch index. This procedure allows the method to use the 2D skeleton to preserve branch topology while using 3D PCA to incorporate spatial geometric orientation. As a result, the estimated growth axis is less likely to be dominated by secondary branches than a global PCA direction computed from the entire point cloud.

The output of this stage is a single normalized growth axis$\;{\hat{d}}_{k}^{N}\;$for each segmented orchid bud instance, expressed in the plane-normalized frame{N}. The estimated axis was then transformed back to the camera frame as:

(36) $${\hat{d}}_{k}^{C}=R_{N}^{C}{\hat{d}}_{k}^{N}$$

The camera-frame vector$\;{\hat{d}}_{k}^{C}\;$is subsequently used to construct the local grasp frame and generate the final robot grasp pose.

### Robot grasp pose generation

After the final unit growth-axis vector$\;{\hat{d}}_{k}^{C}\;$of thek-th orchid bud instance had been obtained in the camera coordinate frame, the next step was to generate a grasp pose for robotic manipulation. The purpose of this stage was to convert the estimated growth axis into a local grasp-oriented coordinate frame and then transform this frame from the camera coordinate system to the robot base coordinate system.

Let$\;g_{k}^{C}\in R^{3}$denote the selected grasp point of thek-th bud expressed in the camera coordinate frame{C}. In this implementation,$\;g_{k}^{C}\;$was selected near the basal region of the reconstructed bud structure because this region provides a more stable geometric reference for grasping or cutting than the terminal region. Before grasp-point computation, the basal point and the required local point-cloud points of thek-th bud were expressed in the camera frame using$\;p_{b,k}^{C}=R_{N}^{C}p_{b,k}^{N}\;$and$\;p_{i,k}^{C}=R_{N}^{C}p_{i,k}^{N}$. The grasp point was computed as the centroid of the local camera-frame point cloud within a small neighborhood around the basal node:

(37) $$g_{k}^{C}=\frac{1}{|N_{b,k}^{C}|}\sum_{p_{i,k}^{C}\in N_{b,k}^{C}}p_{i,k}^{C}$$

where$\;N_{b,k}^{C}\;$is the set of camera-frame 3D points located within a radius$\;r_{b}\;$from the camera-frame basal point$\;p_{b,k}^{C}$. In this study,$\;r_{b}\;$was set to 8 mm.

All axes of the local grasp frame were expressed in the camera coordinate frame{C}. The local grasp frame was constructed by aligning one axis of the gripper with the estimated growth direction. The localz-axis of the grasp frame was defined as:

(38) $$z_{g}^{C}={\hat{d}}_{k}^{C}$$

To construct the remaining orthogonal axes, a camera-frame reference vector$\;r^{C}$, defined as the positivez-axis of the camera coordinate frame, was selected. The localx-axis was calculated as:

(39) $$x_{g}^{C}=\frac{r^{C}\times z_{g}^{C}}{∥r^{C}\times z_{g}^{C}∥}$$

and the localy-axis was then obtained as:

(40) $$y_{g}^{C}=z_{g}^{C}\times x_{g}^{C}$$

If$\;r^{C}\;$was nearly parallel to$\;z_{g}^{C}$, another camera-frame reference vector was selected to avoid numerical instability in the cross-product operation. The resulting rotation matrix of the grasp frame expressed in the camera coordinate system is:

(41) $$R_{g}^{c}=\left[\begin{array}{ccc} x_{g}^{C} & y_{g}^{C} & z_{g}^{C} \end{array}\right]$$

The grasp pose in the camera coordinate system is then expressed as:

(42) $$T_{g}^{c}=\left[\begin{array}{cc} R_{g}^{c} & g_{k}^{C}\\ 0_{1\times 3} & 1 \end{array}\right]$$

To execute the grasp pose with the robot, the pose must be transformed from the camera coordinate system to the robot base coordinate system. Let the camera-to-robot transformation be:

(43) $$T_{c}^{b}=\left[\begin{array}{cc} R_{c}^{b} & t_{c}^{b}\\ 0_{1\times 3} & 1 \end{array}\right]$$

where$\;R_{c}^{b}\;$and$\;t_{c}^{b}\;$describe the rotation and translation of the camera frame with respect to the robot base frame. In the experimental setup, the RGB-D camera was mounted in a fixed eye-to-hand configuration above the workspace. The transformation$\;T_{c}^{b}\;$was obtained before the experiment using an eye-to-hand calibration procedure with a checkerboard target and was kept constant during data acquisition and manipulation. The final robot-referenced grasp pose is:

(44) $$T_{g}^{b}=T_{c}^{b}T_{g}^{c}$$

where$\;T_{g}^{b}\;$represents the 6-DoF grasp pose expressed in the robot base coordinate system. This pose provides the end-effector position and orientation required for perception-guided robotic manipulation of the orchid bud.

**Fig. 9:**
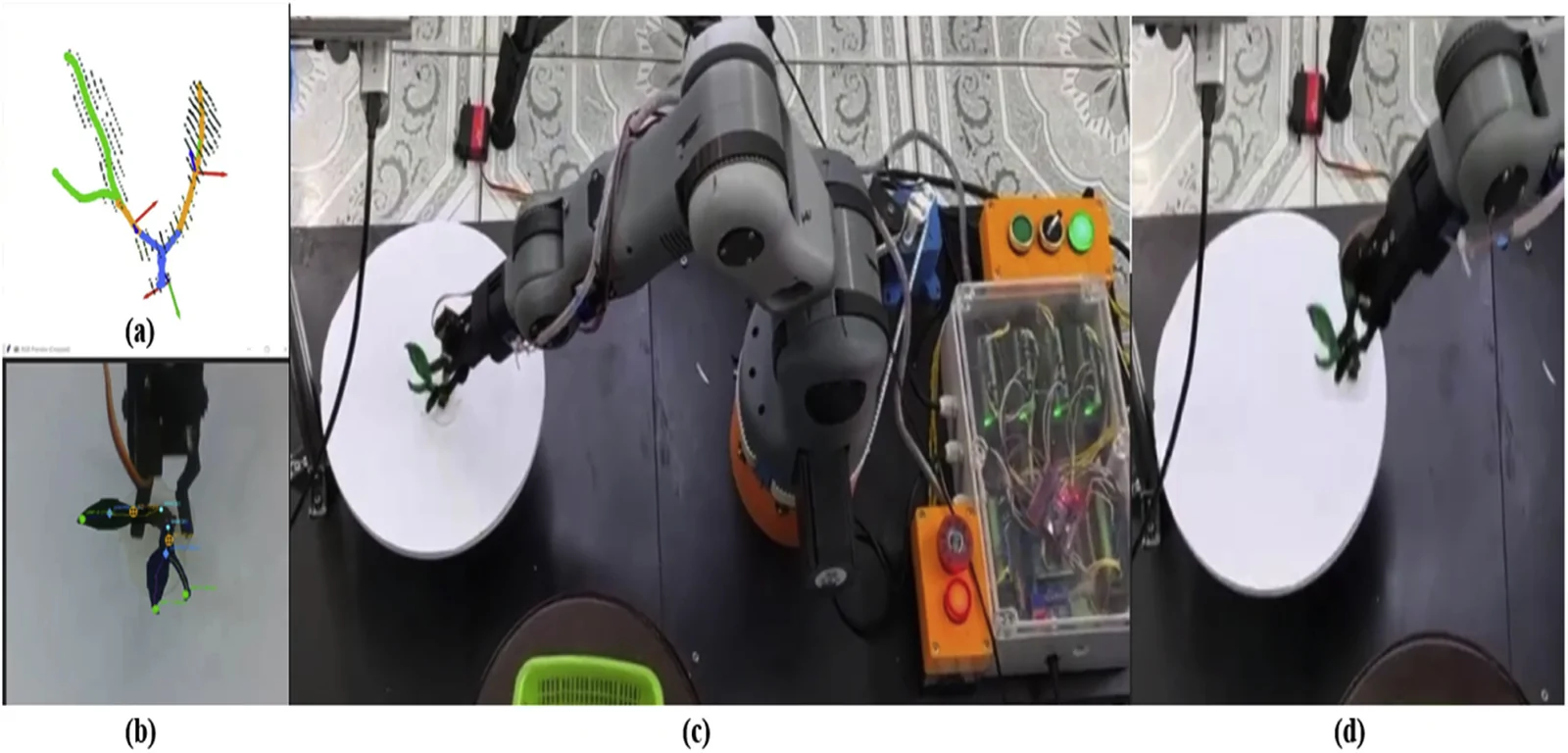
Representative robotic grasping sequence using the generated robot-referenced grasp pose. (a) Estimated growth axis and grasp frame; (b) generated grasp pose projected on the RGB image; (c) robotic approach and grasping near the basal region; and (d) lifting of the orchid bud. This sequence illustrates the conversion of the RGB-D-based growth-axis estimate into an executable robot-referenced grasp pose.

A representative robotic grasping sequence using the generated grasp pose is shown in Fig. 9. The gripper was aligned with the estimated growth axis, approached the basal region, grasped the target, and lifted the orchid bud. Quantitative robotic grasping results are reported in Table 6.

### Implementation and reproducibility settings

The complete RGB-D processing pipeline was implemented in Python 3.11.9. The software environment included Ultralytics 8.3.59 using the YOLOv8s-seg architecture, PyTorch 2.1.2+cu121, torchvision 0.16.2+cu121, OpenCV 4.10.0.84, Open3D 0.19.0, NumPy 1.26.4, scikit-image 0.24.0, pyrealsense2 2.55.1.6486, and SciPy 1.13.1. The installed PyTorch binary used the CUDA 12.1 runtime and cuDNN 8.8.1. The Ultralytics training configuration used a random seed of 0. The Open3D random seed was also fixed at 0 before RANSAC-based supporting-plane estimation to ensure reproducible random sampling. Statistical analyses were conducted using R version 4.6.1 in RStudio 2026.07.1 Build 147. The R environment included lme4 2.0.6, lmerTest 3.2.1, pbkrtest 0.5.5, emmeans 2.0.4, readxl 1.5.0, dplyr 1.2.1, tidyr 1.3.2, ggplot2 4.0.3, and writexl 1.5.4. The train, validation, and test partitions were fixed at the original-image level before data augmentation and were reused unchanged throughout model training and evaluation.

### Method validation

The proposed method was validated using RGB-D data collected from Phalaenopsis orchid buds under controlled imaging and manipulation conditions. An Intel RealSense D435 camera was used to acquire synchronized RGB images and depth maps from a fixed eye-to-hand configuration above the workspace. Before point cloud reconstruction, the depth image was aligned to the RGB image so that each segmented RGB pixel corresponded to the correct depth value.

The validation dataset consisted of 520 RGB-D samples collected from 250 Phalaenopsis orchid bud specimens. The dataset included buds with different orientations and branching patterns, including both single-branch and multi-branch structures. The RGB images had a resolution of 1280 × 720, and the depth maps had a resolution of 1280 × 720. The camera was mounted approximately 60 cm above the workspace. These data were used to evaluate whether the proposed hybrid 2D–3D pipeline could provide more reliable growth axis estimation than methods relying only on 2D topology or only on 3D point cloud geometry.

For the segmentation stage, the instance segmentation model was trained on annotated orchid bud images and then used to generate the masks required for skeleton extraction and point cloud reconstruction. The resulting segmentation model achieved a mask mAP@0.5 of 0.970 on the validation set. Since the main objective of this study is growth axis estimation and grasp pose generation rather than segmentation benchmarking, this result is reported only to confirm that the generated masks were sufficiently reliable for downstream processing. To validate growth axis estimation, three methods were compared: (1) a 2D skeleton-based method, (2) a 3D PCA-based method, and (3) the proposed hybrid 2D–3D fusion method.

The 2D skeleton-based method estimated the dominant base-to-tip direction from the selected skeleton path. For camera-referenced angular evaluation, the basal and terminal skeleton pixels were deprojected into 3D camera coordinates using the aligned depth map and the camera intrinsic parameters, and the resulting unit direction was expressed in the camera frame. The 3D PCA-based method estimated the dominant direction using PCA applied to the reconstructed bud point cloud. The resulting global PCA direction was transformed from the plane-normalized frame{N}to the camera frame{C}using$\;R_{N}^{C}\;$before angular-error calculation. The proposed method combined branch-wise PCA directions with skeleton-derived structural directions as described in the previous sections.

The reference growth axis was manually annotated for each RGB-D sample included in the angular-error evaluation using the aligned RGB and depth images. For multi-branch samples, the reference branch was defined as the continuous basal-to-terminal branch having the greatest visible centerline length from the basal attachment region to a terminal tip. The basal point was selected at the visible branch-attachment region, and the terminal point was selected at the outermost tip of the same branch. Manual annotation was conducted independently of the evaluated algorithms: only the basal and terminal points were marked, without access to any algorithm-generated growth-axis estimates or angular-error results. Both points were marked in image coordinates. If the aligned depth value at an annotated pixel was invalid, the median of the valid depth values within a5×5-pixel neighborhood centered at that point was used. A sample was retained for angular-error evaluation only when a valid depth value could be obtained for both annotated endpoints. Based on this criterion, the final evaluation dataset comprised 520 RGB-D samples. The annotated points were converted into 3D camera coordinates using the corresponding depth values and the RealSense camera intrinsic parameters. The resulting 3D points were visually verified on the reconstructed point cloud solely to confirm that they were located on the manually selected branch before the reference direction was calculated. In a separate automated processing stage, the three evaluated methods generated their corresponding growth-axis estimates, which were subsequently compared with the manually defined reference axes.

For each bud sample, the reference vector was defined as:

(45) $$d_{\text{ref}}^{C}=\frac{p_{\text{tip}}^{C}-p_{\text{base}}^{C}}{∥p_{\text{tip}}^{C}-p_{\text{base}}^{C}∥}$$

where$\;p_{\text{base}}^{C}\;$and$\;p_{\text{tip}}^{C}\;$are the annotated 3D basal and terminal points of the dominant bud branch, respectively. The angular error between the estimated growth axis$\;{\hat{d}}^{C}\;$and the reference direction$\;d_{\text{ref}}^{C}\;$was calculated as:

(46) $$e_{\theta }=\frac{180}{\pi }\text{co}s^{-1}\left(\frac{{\left({\hat{d}}^{C}\right)}^{T}d_{\text{ref}}^{C}}{∥{\hat{d}}^{C}∥∥d_{\text{ref}}^{C}∥}\right)$$

where$\;e_{\theta }\;$is the angular error expressed in degrees. Because the geometric evaluation concerned the orientation of the growth axis rather than its signed base-to-tip direction, the absolute inner product was used so that$\;{\hat{d}}^{C}\;$and$\;-{\hat{\;d}}^{C}$were treated as equivalent axis orientations. The base-to-tip sign required for grasp-pose generation was determined separately from the basal-to-terminal direction of the skeleton branch.

Each RGB-D sample was evaluated using all three estimation methods, resulting in three paired angular-error observations for each sample. Moreover, multiple RGB-D samples could originate from the same physical orchid-bud specimen. The observations were therefore not treated as statistically independent.

Let *y_ijk_* denote the angular error obtained for specimen *i*, RGB-D sample *j* nested within specimen *i*, and estimation methodk. The square-root-transformed angular errors were analyzed using the following linear mixed-effects model:

(47) $$\sqrt{y_{ijk}}=\beta_{0}+\beta_{k}^{\left(M\right)}+\beta_{c_{i}}^{\left(C\right)}+\beta_{k,c_{i}}^{\left(MC\right)}+u_{i}+v_{ij}+\varepsilon_{ijk}$$

where *i=1,…,250* indexes the physical orchid-bud specimens,jindexes the 520 RGB-D samples nested within the specimens, andkindexes the three estimation methods: Hybrid 2D–3D, Global 3D PCA, and 2D Skeleton. The variable$\;c_{i}\;$denotes the branch-complexity category of specimeni, classified as either single-branch or multi-branch. The term$\;\beta_{0}\;$is the intercept;$\;\beta_{k}^{\left(M\right)}\;$represents the fixed effect of estimation method;$\;\beta_{c_{i}}^{\left(C\right)}\;$represents the fixed effect of branch complexity; and$\;\beta_{k,c_{i}}^{\left(MC\right)}\;$represents the interaction between estimation method and branch complexity. The terms$\;u_{i}\;$and$\;v_{ij}\;$denote the random intercepts for specimeniand RGB-D samplejnested within specimeni, respectively, while$\;\varepsilon_{ijk}\;$is the residual error. The random effects and residual errors were assumed to follow mutually independent zero-mean normal distributions:

(48) $$u_{i}∼N\left(0,\sigma_{u}^{2}\right),\;v_{ij}∼N\left(0,\sigma_{v}^{2}\right),\;\varepsilon_{ijk}∼N\left(0,\sigma^{2}\right)$$

Method, branch complexity, and their interaction were included as fixed effects. Random intercepts for RGB-D samples nested within specimens accounted for the paired method observations within each sample and the clustering of multiple samples within the same specimen. Type III fixed-effect tests were conducted using the Kenward–Roger approximation for denominator degrees of freedom. Overall estimated marginal means were equally averaged across the two branch-complexity levels and re-gridded to the original degree scale. Pairwise contrasts were then calculated on the response scale using delta-method standard errors. The correspondingp-values were adjusted using the Holm procedure, whereas the reported 95% confidence intervals were pointwise intervals. Model adequacy was evaluated by examining convergence, singularity, normal Q–Q plots of the residuals, residual histograms, and residual-versus-fitted plots. Statistical analyses were performed in R using the lme4, lmerTest, pbkrtest, and emmeans packages.

As part of method validation, one-factor-at-a-time sensitivity analyses were conducted to characterize the effects of the skeleton neighborhood threshold$\;r_{s}\;$and the fusion weightαon growth-axis estimation accuracy and to establish the default settings used in the reported implementation. During each analysis, only the parameter under investigation was varied, while all other processing parameters and evaluation conditions were kept unchanged. The same RGB-D samples were processed under all tested settings to ensure that the observed differences resulted from parameter variation rather than differences in sample composition. These analyses were intended to assess robustness within the tested parameter ranges rather than to establish globally optimal parameter values.

To evaluate the influence of$\;r_{s}\;$without preferentially weighting either directional cue,αwas provisionally fixed at 0.5. This setting assigns equal nominal weights to the branch-wise PCA direction and the skeleton-derived direction before normalization, thereby providing a neutral fusion condition for isolating the effect of the branch-support neighborhood. The value of$\;r_{s}\;$was then varied from 2 to 11 pixels at 1-pixel intervals. This range was selected to cover both narrow and relatively broad support neighborhoods around the skeleton paths while preserving branch-specific locality. Values greater than 11 pixels were not included because excessively wide neighborhoods would increase the likelihood of overlap between adjacent branch regions and introduce unrelated 3D points into the branch-specific point clouds. Such contamination could reduce the discriminability between neighboring branches and bias the local PCA direction rather than provide additional useful geometric information.

Table 1 summarizes the sensitivity of the growth-axis estimation method to the skeleton neighborhood threshold$\;r_{s}$. For each value of$\;r_{s}$, the mean angular error, standard deviation of angular error, valid branch rate, and overlapping support rate were evaluated using the same RGB-D samples. The valid branch rate was defined as the percentage of candidate branches whose branch-specific point clouds contained at least 10 valid 3D points after depth validation and point-cloud filtering and yielded a finite, non-zero principal direction through PCA. The overlapping support rate was defined as the percentage of valid foreground pixels located within the$\;r_{s}$-neighborhoods of two or more candidate skeleton paths before nearest-path assignment.

As shown in Table 1, increasing$\;r_{s}\;$from 2 to 5 pixels reduced the mean angular error from 7.8° to 5.3° and increased the valid branch rate from 91.0% to 99.0%. Small$\;r_{s}\;$values produced sparse branch-specific point clouds, resulting in less stable PCA directions. Within the tested range, the lowest mean angular error was observed at$\;r_{s}=5\;$pixels, with a standard deviation of 3.4°, a valid branch rate of 99.0%, and an overlapping support rate of 3.0%. Increasing$\;r_{s}\;$beyond 5 pixels produced only marginal gains in the valid branch rate while progressively increasing overlap between adjacent branch-support regions, reaching 23.0% at$\;r_{s}=11\;$pixels. This increased overlap reduced branch-specific locality and introduced points associated with neighboring branches into the local PCA estimation. Therefore,$\;r_{s}=5\;$pixels was adopted as the default setting for the reported implementation because it provided the most favorable balance among estimation accuracy, valid branch coverage, and branch separation within the tested range.

After selecting the skeleton neighborhood threshold,$\;r_{s}\;$was fixed at 5 pixels, and the influence of the fusion weightαwas evaluated. The value ofαwas varied from 0.0 to 1.0 at intervals of 0.1. This range covers the two limiting cases, whereα=0represents the skeleton-derived direction alone andα=1represents the branch-wise PCA direction alone, as well as intermediate combinations of the two directional cues. All other processing parameters and evaluation conditions were kept unchanged. To examine whether the preferred fusion weight depended on branch complexity, the angular error was evaluated for the complete dataset and separately for single-branch and multi-branch bud instances.

Accordingly, the error of 5.3° reported for$\;r_{s}=5\;$in Table 1 corresponds to the provisional settingα=0.5. After$\;r_{s}\;$was fixed at 5 pixels,α=0.6was adopted as the default setting based on the subsequent fusion-weight analysis, resulting in a final descriptive mean angular error of$\;5.1\pm 3.3^{\circ }$.

Table 2 summarizes the influence of the fusion weightαon growth-axis estimation accuracy. For each tested value, the mean angular error and standard deviation were calculated for the complete evaluation dataset and separately for single-branch and multi-branch bud structures. This analysis focused on estimation accuracy because varyingαonly changes the final weighted combination of the previously computed PCA-based and skeleton-derived directions, without altering the preceding processing stages.

The limiting settingsα=0andα=1should not be interpreted as identical to the standalone 2D skeleton and global 3D PCA baselines, respectively. Atα=0, the skeleton-derived direction was still mapped into 3D space using aligned depth information, whereas atα=1, PCA was applied to skeleton-guided branch-specific point clouds rather than to the complete bud point cloud. All preceding branch extraction and 3D processing stages therefore remained active.

As shown in Table 2, increasingαfrom 0.0 to 0.6 progressively reduced the overall mean angular error from 9.0° to 5.1°. Within the tested range, the lowest overall mean error was observed atα=0.6, with corresponding mean errors of 4.3° and 6.2° for single-branch and multi-branch structures, respectively. The overall mean error remained within a narrow range of 5.1°–5.3° forα=0.5–0.7, indicating that the method was locally robust to moderate variations around the selected value. Whenαexceeded 0.7, the increasing reliance on branch-wise point-cloud geometry resulted in larger errors, particularly for multi-branch structures. Therefore,α=0.6was adopted as the default fusion weight for the reported implementation.

**Fig. 10:**
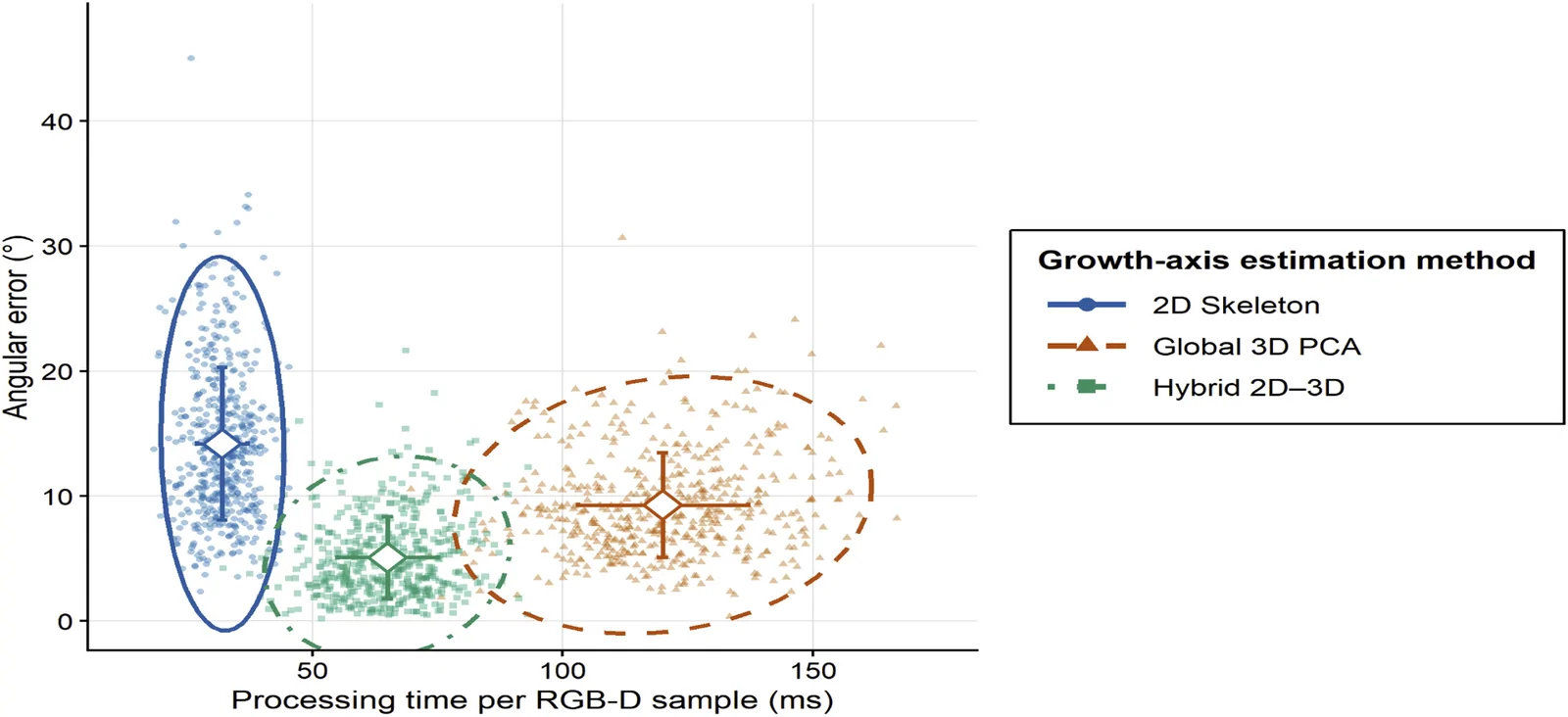
Processing-time–angular-error trade-off for the three growth-axis estimation methods. Points represent individual RGB-D samples; diamonds indicate method-specific means, error bars indicate ±1 SD, and ellipses show 95% covariance regions.

Table 3 summarizes the raw descriptive performance of the three evaluated methods. The Hybrid 2D–3D method achieved the lowest raw mean angular error of 5.1°, compared with 9.3° for Global 3D PCA and 14.2° for the 2D Skeleton method. The corresponding standard deviations were 3.3°, 4.2°, and 6.1°, respectively. In terms of computational efficiency, the Hybrid 2D–3D method required an average processing time of 65 ms per RGB-D sample, compared with 32 ms for the 2D Skeleton method and 120 ms for Global 3D PCA. These descriptive results indicate that the Hybrid 2D–3D method substantially reduced angular error while remaining faster than the Global 3D PCA baseline. All processing-time measurements reported in Table 3 and Fig. 10 were obtained on a workstation equipped with an AMD EPYC 7H12 64-Core Processor, two NVIDIA GeForce RTX 4090 GPUs, and 256 GB of RAM.

**Table 3:** Unadjusted sample-level descriptive performance of the three growth-axis estimation methods.

**Growth-axis estimation method**	**Angular error, mean ± SD (°)**	**Mean processing time per RGB-D sample (ms)**
2D Skeleton	14.2 ± 6.1	32
Global 3D PCA	9.3 ± 4.2	120
Hybrid 2D-3D	5.1 ± 3.3	65

Note: Angular-error values are unadjusted arithmetic means ± standard deviations based on 520 RGB-D samples collected from 250 physical specimens. Processing time is reported as the mean time per RGB-D sample. Inferential comparisons accounting for paired measurements and specimen-level clustering are reported in Table 4, Table 5.

The trade-off between angular error and computational cost is further illustrated in Fig. 10. The 2D Skeleton method achieved the shortest processing time but the highest angular error, whereas Global 3D PCA required the longest processing time. The Hybrid 2D–3D method achieved the lowest angular error while remaining substantially faster than Global 3D PCA, indicating a favorable balance between estimation accuracy and computational efficiency.

Because each RGB-D sample was evaluated using all three methods and multiple samples could originate from the same physical specimen, inferential comparisons were performed using the linear mixed-effects model described above. This analysis accounted for both the paired observations within each RGB-D sample and the clustering of multiple samples within specimens. The fixed-effect tests and Holm-adjusted pairwise method contrasts are reported below.

**Fig. 11:**
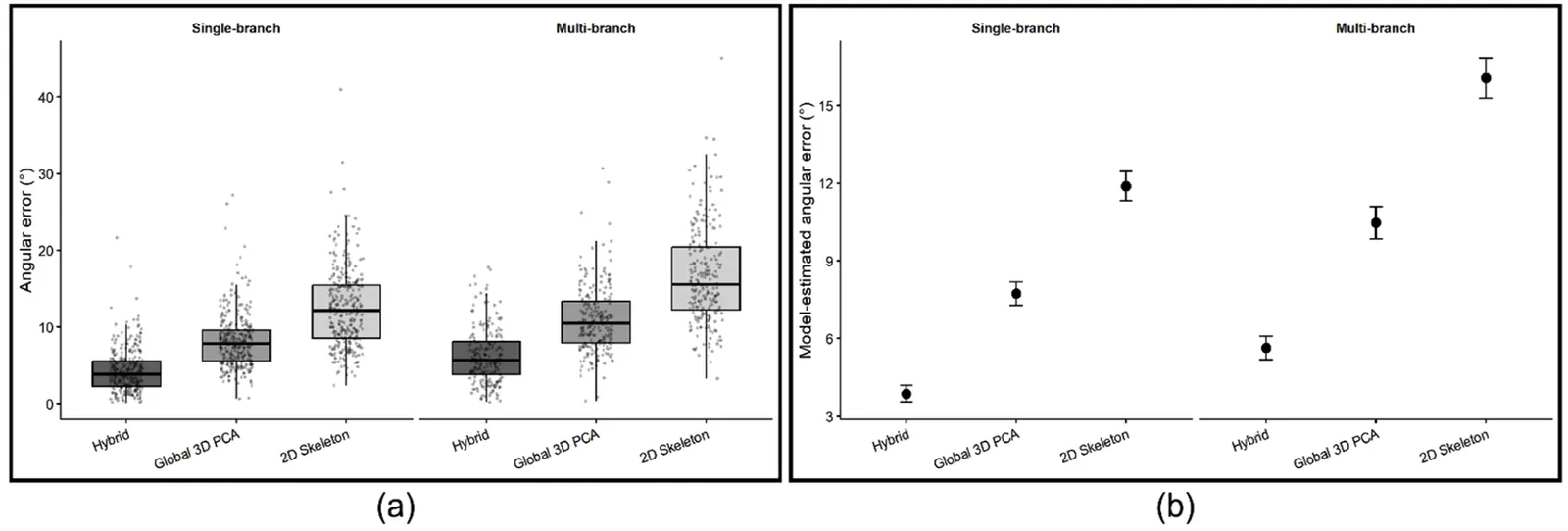
Descriptive and model-based comparisons of growth-axis estimation errors across branch complexities. (a) Raw sample-level angular-error distributions for the Hybrid 2D–3D, Global 3D PCA, and 2D Skeleton methods. Individual points represent angular-error observations from RGB-D samples, boxes indicate the interquartile range, and horizontal lines indicate the median. (b) Back-transformed model estimates and pointwise 95% confidence intervals obtained from the linear mixed-effects model.

The raw angular-error distributions stratified by branch complexity are presented in Fig. 11(a), whereas the corresponding back-transformed model estimates and pointwise 95% confidence intervals are shown in Fig. 11(b). Across both single-branch and multi-branch samples, the Hybrid 2D–3D method consistently exhibited the lowest angular errors, followed by Global 3D PCA and the 2D Skeleton method. Angular errors were generally higher for multi-branch samples than for single-branch samples for all three methods.

For single-branch samples, the raw mean angular errors were 4.3 ± 2.8°, 8.1 ± 3.6°, and 12.4 ± 5.2° for the Hybrid 2D–3D, Global 3D PCA, and 2D Skeleton methods, respectively. The corresponding values for multi-branch samples were 6.2 ± 3.6°, 10.9 ± 4.4°, and 16.7 ± 6.4°, respectively. These descriptive results indicate that angular errors were generally higher for multi-branch samples than for single-branch samples, while the ordering of the three methods remained consistent across both branch-complexity groups.

The linear mixed-effects analysis revealed significant main effects of estimation method,F(2,1036)=820.51,p<0.001, and branch complexity,F(1,246)=102.28,p<0.001. The method-by-branch-complexity interaction was not significant,F(2,1036)=2.09,p=0.124. Thus, angular error differed among the three methods and was generally higher for multi-branch samples, whereas the relative differences among methods did not vary significantly with branch complexity.

Because the method-by-branch-complexity interaction was not statistically significant, the primary method comparisons were averaged equally across the two complexity levels. The back-transformed estimated marginal means were 4.71° for the Hybrid 2D–3D method, 9.05° for Global 3D PCA, and 13.88° for the 2D Skeleton method. Holm-adjusted pairwise comparisons showed that the Hybrid 2D–3D method produced significantly lower angular errors than both Global 3D PCA and the 2D Skeleton method, while Global 3D PCA also outperformed the 2D Skeleton method (allp<0.001; Table 5).

**Table 4:** Fixed-effect tests from the linear mixed-effects model for angular error.

**Effect**	**Numerator df**	**Denominator df**	F**value**	p**value**
Method	2	1036	820.51	< 0.001
Branch complexity	1	246	102.28	< 0.001
Method × branch complexity	2	1036	2.09	0.124

Note: Angular error was square-root transformed before analysis. Denominator degrees of freedom were calculated using the Kenward–Roger approximation. Random intercepts were included at the specimen level and at the RGB-D sample-within-specimen level.

**Table 5:** Holm-adjusted pairwise comparisons of estimated marginal mean angular error between methods.

(A) Back-transformed estimated marginal mean (°) **Method**	**Estimated marginal mean (°)**	**SE**	**df**	**Pointwise 95% CI (°)**		
Hybrid 2D–3D	4.71	0.140	743.69	4.44 – 4.99		
Global 3D PCA	9.05	0.194	743.69	8.67 – 9.43		
2D Skeleton	13.88	0.240	743.69	13.41 – 14.35		
(B) Pairwise method comparisons with Holm-adjustedp-values						
**Comparison**	**Estimated difference (°)**	**SE**	**df**	**Pointwise 95% CI (°)**	t**ratio**	**Holm-adjusted**p
Hybrid 2D–3D - Global 3D PCA	−4.33	0.204	743.69	−4.73 to −3.93	−21.291	< 0.001
Hybrid 2D–3D - 2D Skeleton	−9.16	0.240	743.69	−9.64 to −8.69	−38.132	< 0.001
Global 3D PCA - 2D Skeleton	−4.83	0.261	743.69	−5.34 to −4.32	−18.490	< 0.001

Note: Panel (A) reports model-based, back-transformed estimated marginal means equally averaged across branch-complexity levels. Panel (B) reports response-scale pairwise contrasts after re-gridding. Confidence intervals are pointwise 95% intervals andp-values are Holm-adjusted; minor discrepancies from subtracting the displayed means reflect rounding because contrasts were computed from unrounded estimates.

**Fig. 12:**
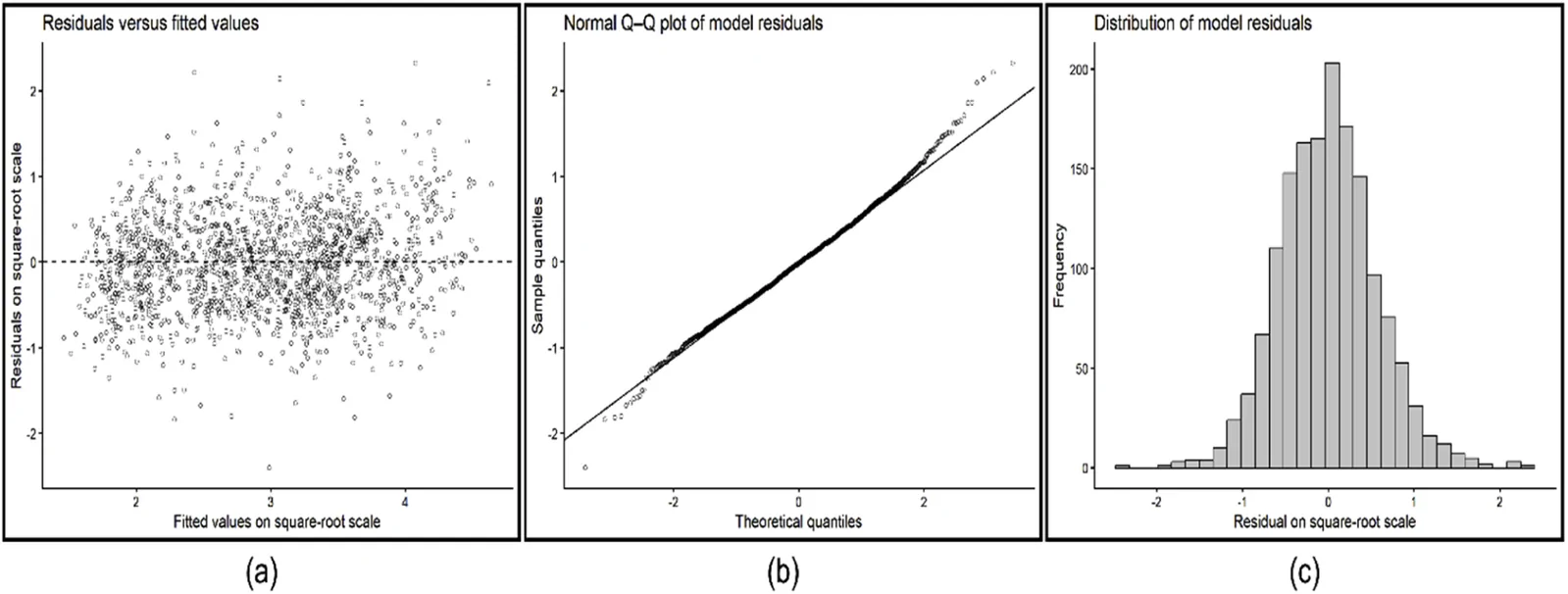
Residual diagnostic plots for the linear mixed-effects model fitted to square-root-transformed angular errors. (a) Residuals versus fitted values on the square-root scale. The dashed horizontal line indicates zero residual. (b) Normal Q–Q plot of the model residuals. (c) Histogram of the model residuals. The diagnostic plots showed moderate deviations in the distribution tails but no pronounced systematic pattern or substantial increase in residual dispersion across fitted values.

The adequacy of the fitted linear mixed-effects model was further evaluated using residual diagnostics. The model showed no evidence of a singular fit and produced no convergence warning. Residual skewness was 0.198, and residual excess kurtosis was 0.723. The correlation between absolute residuals and fitted values was low (r=0.070), indicating no substantial increase in residual dispersion across the fitted-value range. Seven of the 1,560 residuals (0.449%) exceeded an absolute scaled-residual value of 3. As shown in Fig. 12, the residual-versus-fitted plot exhibited no pronounced systematic or funnel-shaped pattern, while the residual histogram was approximately symmetric. The normal Q–Q plot showed moderate deviations in the distribution tails, but the majority of residuals closely followed the reference line. Overall, the diagnostic results were considered acceptable for the fitted model.

To quantitatively evaluate the practical applicability of the generated grasp poses, 50 real robotic grasping attempts were conducted under controlled laboratory conditions using 22 physical orchid-bud specimens. Nine single-branch specimens were used for 20 grasp attempts, whereas 13 multi-branch specimens were used for 30 grasp attempts. Some specimens were tested two or three times. Before each repeated attempt, the specimen was repositioned to provide a different bud orientation relative to the camera and robot, and the complete perception and grasp-pose generation pipeline was executed again.

The experiments used a custom gripper fabricated from PETG by three-dimensional printing. The gripper was a prototype and had not yet been fully optimized for compliant handling of delicate orchid tissues. For each attempt, the complete perception pipeline was used to estimate the growth axis, generate the robot-referenced grasp pose, and command the gripper to approach, grasp, and lift the target.

A grasp attempt was classified as successful when the target was lifted by 20 cm and maintained at that height for at least 5 s without slipping or visible tissue damage. Visible tissue damage was recorded when cracking or breakage of the orchid bud could be identified by unaided visual inspection.

As shown in Table 6, 47 of the 50 robotic grasping attempts resulted in successful lifting without slipping or visible tissue damage, corresponding to an overall attempt-level success rate of 94.0%. The single-branch and multi-branch success rates were 95.0% and 93.3%, respectively. One multi-branch attempt failed because of slipping during lifting. Visible tissue damage, defined as visually identifiable cracking or breakage, occurred in one single-branch attempt and one multi-branch attempt. These damage cases may have been associated with the geometry and contact surfaces of the prototype PETG gripper, which had not yet been fully optimized for handling delicate orchid tissues. Further optimization of the fingertip geometry, contact material, gripping force, and mechanical compliance is therefore required.

**Table 6:** Summary of robotic grasping trials and success outcomes.

**Bud structure**	**Grasp attempts**	**Successful lifts without slipping or visible damage**	**Success rate (%)**	**Slipping failures**	**Visible tissue damage**
Single branch	20	19	95.0	0	1
Multi branch	30	28	93.3	1	1
**Overall**	**50**	**47**	**94.0**	**1**	**2**

In addition to the quantitative results summarized in Table 6, Fig. 9 presents a representative robotic grasping sequence using the generated grasp pose. The robot aligned the gripper with the estimated growth axis, approached the orchid bud near the basal region, closed the gripper, and lifted the target. Together, the quantitative trials and the representative robotic execution demonstrate that the proposed pipeline can transform RGB-D-based growth-axis estimates into executable robot-referenced grasp poses under controlled laboratory conditions. Nevertheless, these experiments provide preliminary validation and do not yet address collision-aware motion planning, contact-force control, or comprehensive optimization of the gripper–tissue interaction.

Overall, the validation results demonstrate that the proposed hybrid method improves growth-axis estimation accuracy and robustness compared with topology-only and geometry-only baselines while maintaining moderate computational cost. The robotic grasping evaluation yielded an attempt-level success rate of 94.0%, further indicating that the estimated growth axis can be converted into an executable robot-referenced grasp pose for downstream agricultural manipulation under controlled laboratory conditions. These findings support the use of the method as a practical and reproducible approach for RGB-D-based orchid bud structure analysis and perception-guided robotic grasp pose generation.

### Limitations

The proposed method has several limitations. First, its performance depends strongly on the quality of the instance segmentation results. Inaccurate masks may affect both 2D skeleton extraction and 3D point cloud reconstruction, and errors introduced at this stage can propagate through the subsequent fusion process. This may reduce the reliability of both growth axis estimation and grasp pose generation.

Second, the geometric accuracy of the reconstructed point cloud is influenced by the quality of the RGB-D data. Depth noise, missing depth values, surface reflections, and partial occlusions may reduce the stability of the estimated 3D orientation, particularly for small, thin, or partially occluded orchid structures. These factors may also affect the accuracy of the selected grasp point and the resulting robot-referenced pose.

Third, although the proposed 2D–3D fusion strategy improves robustness in multi-branch structures, the method may still be challenged by severe overlap, highly complex branching patterns, or ambiguous structural boundaries. In such cases, distinguishing the dominant growth branch from nearby secondary branches may become less stable, which can influence the consistency of the generated local grasp frame.

Fourth, the current grasp pose generation step is based primarily on geometric and topological cues derived from visual perception. It does not explicitly model contact mechanics, gripper–object interaction, deformation of plant tissues, or collision constraints with neighboring structures. Therefore, although the generated poses were executable in the controlled robotic experiments reported here, they should not be interpreted as fully optimized manipulation solutions under general operating conditions.

Fifth, the current validation was conducted on Phalaenopsis orchid buds under relatively controlled imaging and manipulation conditions. Further evaluation is needed under greenhouse conditions, variable lighting, denser plant scenes, and more complex robotic operation settings.

In addition, the custom gripper used in the present experiments was fabricated from PETG using three-dimensional printing and was not specifically optimized for compliant contact with delicate orchid tissues. Its relatively rigid contact surfaces may increase localized pressure during grasp closure; therefore, future work should investigate softer contact materials, compliant fingertip structures, and controlled gripping force.

## Ethics statements

This study involved RGB-D imaging, structural analysis, and robotic manipulation experiments on Phalaenopsis orchid buds under controlled laboratory conditions. It did not involve human participants, animal experiments, or data collected from social media platforms. Therefore, ethical approval and informed consent were not required.

## CRediT author statement

**Tien-Duc Nguyen:** Methodology, Software, Data curation, Validation, Formal analysis, Visualization, Writing – original draft. **Minh-Hieu Pham:** Software, Investigation, Data curation, Validation, Visualization, Writing – original draft. **Minh-Hau Le:** Software, Investigation, Data curation, Formal analysis, Visualization. **Hoang-Hiep Ly:** Methodology, Formal analysis, Validation, Writing – review & editing. **Xuan-Thuan Nguyen:** Conceptualization, Methodology, Writing – review & editing. **Thi-Thoa Mac:** Conceptualization, Supervision, Writing – review & editing. **Hong-Hai Hoang:** Conceptualization, Methodology, Supervision, Project administration, Funding acquisition, Writing – review & editing.

## Declaration of interests

The authors declare that they have no known competing financial interests or personal relationships that could have appeared to influence the work reported in this paper.

## Data Availability

Code, weights, data and calibration are available: https://github.com/Hieuthichcode/automated-orchid-propagation-system; https://drive.google.com/drive/folders/1FqTuIMWtxHYL037exPB-O3m-0CBoGs5N.
